# Role of Tailored Timing and Frequency Prompts on the Efficacy of an Internet-Delivered Stress Recovery Intervention for Health Care Workers: Randomized Controlled Trial

**DOI:** 10.2196/62782

**Published:** 2025-01-28

**Authors:** Auguste Nomeikaite, Odeta Gelezelyte, Maria Böttche, Gerhard Andersson, Evaldas Kazlauskas

**Affiliations:** 1 Center for Psychotraumatology Institute of Psychology Vilnius University Vilnius Lithuania; 2 Division of Clinical Psychological Intervention Department of Education and Psychology Freie Universität Berlin Berlin Germany; 3 Department of Behavioural Sciences and Learning Linköping University Linköping Sweden; 4 Department of Clinical Neuroscience Karolinska Institutet Stockholm Sweden; 5 Department of Biomedical and Clinical Sciences Linköping University Linköping Sweden

**Keywords:** internet interventions, mental health, stress, health care workers, short message service, cognitive behavioral therapy, internet-delivered cognitive behavioral therapy, psychotherapy, randomized, controlled trial, engagement, SMSl worker, usage, occupational health, provider, prompt, message

## Abstract

**Background:**

Prompts offer a promising strategy to promote client engagement in internet-delivered cognitive behavioral therapy (ICBT). However, if the prompts do not meet the needs of clients, they can potentially be more obtrusive rather than helpful.

**Objective:**

The aim of this study was to test if prompts tailored based on timing and frequency, aligned with preintervention goal setting, can increase usage and the efficacy of a therapist-supported ICBT stress recovery intervention for health care workers.

**Methods:**

The 2-arm randomized controlled trial included 87 health care workers (99% female, aged 19-68 years: mean 39.61, SD 11.49): 43 in the standard intervention group and 44 in the tailored prompts group. The primary outcome measure was the Recovery Experiences Questionnaire, and the secondary outcomes were the Perceived Stress Scale-4, the Patient Health Questionnaire-4, and the World Health Organization-5 Well-Being Index. The self-report data were collected before the intervention (September 2022), postintervention (October 2022), and 6-month follow-up (May 2023).

**Results:**

The results showed that tailored prompts, although appreciated by the majority (39/40, 98%), did not improve intervention usage indicators, such as the number of logins (*t*_85_=–0.91; *P*=.36), modules opened (*t*_83.57_=–1.47; *P*=.15), modules completed (*t*_85_=–0.71; *P*=.48), exercises completed (*t*_85_=–1.05; *P*=.30), or the time spent using the program (*χ*^2^_2_=1.1; *P*=.57). Similarly, tailored prompts did not increase the effects of the intervention in terms of stress recovery skills (Cohen *d* ranging from 0.31 to 0.85), perceived stress (*d*=–0.08; –0.70), depression (*d*=–0.11; –0.38), anxiety (*d*=–0.32; –0.64), or psychological well-being (*d*=0.26; 0.46). In addition, the standard intervention group showed greater long-term stress recovery effects than the group using the internet-delivered intervention supplemented by tailored prompts (β=–0.24, *P*=.03).

**Conclusions:**

Although the study confirmed the efficacy of the program, the merits of tailored prompts in ICBT for stress recovery were not supported. Future research is needed to test the effects of the stress recovery intervention supplemented by goal setting and tailored prompts.

**Trial Registration:**

ClinicalTrials.gov NCT05553210; https://clinicaltrials.gov/study/NCT05553210

## Introduction

### Background

Health care workers (HCWs) are at risk of stress, burnout, and other mental health problems [1,2]. However, long working hours, night shifts, rigid schedules, and prevailing stigma can make it difficult for them to engage in traditional psychological treatment [3]. Internet-delivered cognitive behavioral therapy (ICBT) could be a viable alternative and has shown efficacy in helping HCWs develop stress recovery skills, reduce stress, anxiety, and depression, and improve overall psychological well-being [4,5]. However, findings in these studies also indicated that only half of the included participants familiarized themselves with the full content of the intervention. A qualitative study of early dropouts revealed that many identified barriers to engagement, such as a lack of time or motivation and unmet expectations or needs [6]. Across a range of diagnoses, better adherence has emerged as a predictor of better outcomes in internet interventions for adults [7]. Thus, it is crucial to find ways to motivate HCWs’ engagement in internet interventions but in a way that considers individual needs and time constraints.

To enhance retention in ICBT, various persuasion techniques, such as text message reminders, have been proposed [8]. The inclusion of prompts to encourage engagement in internet interventions for healthy behavior and mental health has shown promising results [9]. Research findings are, however, inconsistent, with studies finding no significant clinical benefits of supplementary prompts in digital treatment [10]. Indeed, if prompts received are not relevant for the user, they can have the opposite effect and be more obtrusive rather than helpful [11]. Tailoring the frequency and timing of prompts could be a potential solution in internet-delivered stress management treatment [12], although there is still scarce knowledge of whether this affects engagement and intervention effects. Another suggested solution to reducing negative emotions caused by reminders is to set goals [13], such as how much time a user intends to spend on treatment. To conclude, setting usage goals and delivering tailored prompts could be a way to promote the engagement of health care workers and consequently increase the efficacy of the internet-delivered stress recovery intervention.

### Objectives

In this study, we aimed to test whether the inclusion of tailored prompts aimed at achieving usage goals can increase the efficacy of an ICBT stress recovery intervention for health care workers in a randomized controlled trial (RCT). The “For Recovery from Stress” (FOREST) is a 6-week ICBT intervention [14], incorporating mindfulness and focused on stress recovery [15]. FOREST+ is the updated version of FOREST, designed to meet the needs of health care workers [5]. The following four objectives were set: (1) to analyze associations between tailored prompts and different engagement indicators in a stress recovery intervention; (2) to assess whether the intervention with tailored prompts is more effective in improving stress recovery skills as compared with the standard intervention; (3) to test if the inclusion of tailored prompts can improve the effects of the internet intervention on stress, anxiety, depression, and psychological well-being; and (4) to explore how having an option to receive tailored prompts alters the users program evaluation.

## Methods

### Study Design

A 2-arm RCT was conducted to investigate how usage goal setting with prompts tailored by timing and frequency would be related to the usage and the efficacy of an internet-delivered intervention for stress recovery. Eligible participants were randomized (1:1) by an independent researcher into 2 study groups: a standard intervention group (SG) or a tailored prompts group (TG), using built-in randomization functionality in the hosting platform. Before registering for the study, participants were informed that the intervention would be provided either with tailored prompts or without. Both groups started using the program after randomization in October 2022. The assessments took place on three occasions: (1) before the intervention (September 2022), (2) post intervention (October 2022), and (3) at the 6-month follow-up (May 2023). Participants’ self-reported data and data on the use of the program were collected using a secure platform*,* Iterapi*,* which hosted the program [16].

### Ethical Considerations

The study was carried out following the local and international ethical regulations for research with human participants. The participants' contact details (phone number and email) collected during the initial assessment were only used for contact purposes of the study and were anonymized for analysis. The study was approved by the Vilnius University Psychology Research Ethics Committee (reference number 2021-03-22/61). The trial has been registered on ClinicalTrials.gov (NCT05553210). Multimedia Appendix 1 contains the study’s CONSORT-EHEALTH (Consolidated Standards of Reporting Trials of Electronic and Mobile Health Applications and Online Telehealth) checklist [17].

### Recruitment and Procedure

The call for participation in the intervention was disseminated nationwide in Lithuania through various social networks, emails to regional and national professional HCWs societies and groups, and health care institutions. Those interested could register for the intervention through the intervention website, which provided detailed information about the program and the research study. After completing the registration form and the preintervention questionnaire, participants were contacted for a brief phone interview to ensure that they met the eligibility criteria for the study which are (1) currently working in a health care facility, (2) being an adult (>18 years), (3) comprehending Lithuanian, and (4) having access to a device with internet connection. Certain exclusion criteria have also been identified which are (1) high risk of suicide, (2) acute psychiatric crisis, and (3) exposure to the current interpersonal violence. Participants who did not meet the eligibility criteria (n=9) were referred elsewhere for psychological support if needed.

### Intervention

**Figure 1:**
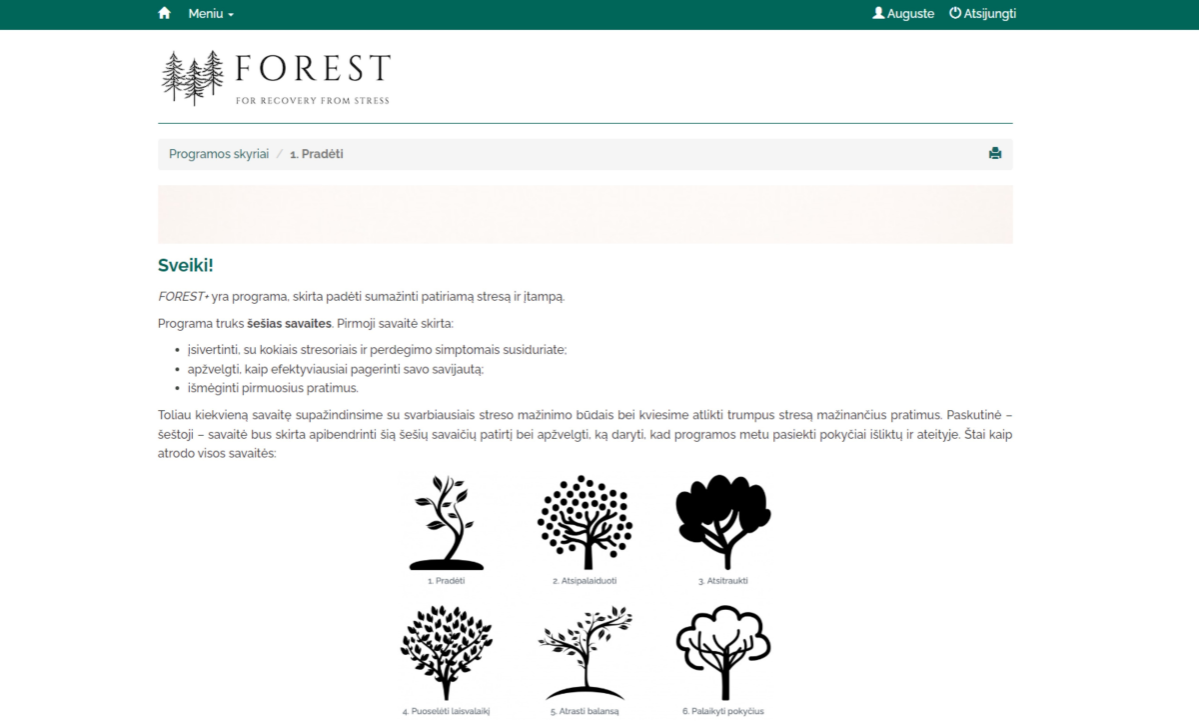
User interface.

FOREST+ is a 6-week stress recovery program specifically designed in close collaboration with health care professionals for health care workers in Lithuania [5]. FOREST+ was developed as a modification of FOREST for nurses [4,14]. The FOREST+ program consists of 6 modules, opened on a weekly schedule which are (1) introduction, (2) psychological detachment, (3) distancing, (4) mastery, (5) control, and (6) keeping the change alive. The modules provide psychoeducational information based on the principles of CBT, mindfulness, and 4 components of stress recovery (mastery, control, psychological detachment, and distancing), as well as various exercises such as mindfulness recordings and self-assessment of bodily tension and stress. The user interface of the program is shown in Figure 1. The language of the program was Lithuanian, and the content of the modules is described in Table 1.

The program is delivered with therapist support using a low-intensity approach. Once the users had completed a weekly worksheet, they received personalized feedback from their therapist. Participants could also contact their therapist through text messaging integrated into the intervention platform. In addition to communication with the therapist, clients received support from administrators through emails to keep them informed about the progress of the program and to remind them to join a new module or complete worksheets. Administrators also called the participants for a short interview before the intervention and in the middle of the program to inform them of the progress and answer any questions they may have on the use of the intervention.

In the TG, client-administrator communication was supplemented by reminders through SMS. Prompts consisted of a short text asking if the participant had already taken the time to unwind and a link to the mindfulness exercises for that week. SMS prompts were tailored to each participant in the group during a phone interview with the study administrator before using the program. Participants were asked how actively they planned to engage with the program and whether they would need short text message reminders to achieve this. When participants indicated that supplementary prompts would be necessary, they were asked about preferences of prompt frequency (ie, once a week, twice a week, every workday, or every workday twice a day) and timing (before noon, sometime in the morning or afternoon, sometime in the evening). Participants in the SG did not schedule a plan for the tailored prompts and did not receive them.

**Table 1:** Description of the program modules.

Module	Description	Exercises, n
1. Introduction	The initial module introduces how to use the program and provides psychoeducational content on occupational stress. Exercises focusing on stressors and symptoms of burnout are included, as well as a short relaxation audio recording for mindful breathing.	4
2. Psychological detachment	The focus is on managing stress through body relaxation and improving sleep quality. An exercise for progressive muscle relaxation is included, as well as exercises to assess the level of tension in the body, along with a recording for relaxation before bedtime.	4
3. Distancing	The module goes further on the importance of detachment from work during leisure time. Exercises alongside the psychoeducational material are designed to identify intrusive thoughts and activities that help distract oneself from thoughts about work. Out of 2 relaxations are included which are one for raising awareness of the present and one for walking meditation.	3
4. Mastery	This module covers another part of stress recovery, nurturing leisure time and a sense of competence outside work. The exercises in this part allow the user to assess the level of physical activity and the activities that help to relax and feel a sense of mastery. A brief relaxation recording and a video of body stretching exercises are included.	3
5. Control	The ways to actively pursue work-life balance are presented in the module, and the importance of feeling in control of one’s time is explained. The exercises are designed to help set daily needs and notice activities that interfere with work-life balance.	3
6. Keeping the change alive	The last module focuses on how to maintain the change after the end of the program. The exercises are designed to review the activities covered during the program and the relaxation exercises that could help maintain work-life balance. Also included is a relaxation recording for overall relaxation.	2

### Measures

#### Demographic Questionnaire

The preintervention measures were used to collect sociodemographic and work-related information. Sociodemographic data were collected by asking questions on gender, age, education, relationship status, experience of psychological treatment or support, and the use of medication for mental health difficulties. Information on work-related aspects such as position, work status, type of service, and work experience was also included.

#### Recovery From Stress

An overall recovery from stress experience was measured by the Recovery Experiences Questionnaire (REQ) [15]. The REQ consists of 16 items (eg, “I take time for leisure”). Each of the items can be evaluated on a 5-point Likert-type scale, where 1 is “Totally Disagree” and 5 is “Totally Agree”; scores are calculated by adding up the points, with higher scores indicating a more pronounced stress recovery experience. The REQ showed good validity in the Lithuanian sample [18]. In this study, the REQ McDonald’s Omega was excellent at all 3 measurements: ω_T1_=0.87, ω_T2_=0.90, ω_T3_=0.88.

#### Perceived Stress

The brief Perceived Stress Scale (PSS-4) [19] was used to measure stress levels. The PSS-4 consists of 4 questions (eg, “In the last month, how often have you felt that you were unable to control the important things in your life?”). The questions are answered on a 5-point Likert-type scale, where 0 is “Never” and 4 is “Very often”; with the final score calculated by summing all the responses (reversing the scores of items 2 and 3). Higher scores show higher levels of perceived stress. Studies show good psychometric properties of the PSS-4 scale [19]. The PSS-4 McDonald’s Omega in the current sample was moderate but close to the acceptable level of 0.70 [20]: ω_T1_=0.67, ω_T2_=0.69, ω_T3_=0.61.

#### Anxiety and Depression

Participants’ levels of anxiety and depression were assessed using the 4-item Patient Health Questionnaire (PHQ-4) [21]. The PHQ-4 consists of 2 items to assess depression (eg, “Feeling down, depressed, or hopeless”) and 2 to assess anxiety (eg, “Feeling nervous, anxious, or on edge”). The respondent rates how much each item bothered them on a 4-point Likert-type scale, where 0 is “Not at all” and 3 is “Nearly every day.” Adding the item scores for each subscale gives an estimated level of depression or anxiety. Research has shown good PHQ-4 psychometric properties in the Lithuanian sample [18]. The PHQ-4 Cronbach alpha in the current sample for the anxiety subscale was acceptable: α_T1_=0.70, α_T2_=0.74, α_T3_=0.78; as well as for the depression subscale: α_T1_=0.71, α_T2_=0.82, α_T3_=0.80.

#### Psychological Well-Being

The psychological well-being was measured with the World Health Organization-5 Well-Being Index (WHO-5). WHO-5 consists of 5 items (eg, “I have felt calm and relaxed”), which are rated on a 6-point Likert-type scale, where 0 is “At no time” and 5 is “All the time.” The final percentage well-being score (ranging from 0 to 100) is calculated by summing the item scores and then multiplying the raw score by 4. Higher final scores indicate better psychological well-being. The WHO-5 has been translated and used in Lithuanian sample studies [22]. The WHO-5 McDonald’s Omega in the current sample was good: ω_T1_=0.83, ω_T2_=0.86, ω_T3_=0.82.

#### Acceptability

The postintervention measurement included questions on the user experience of the program. Participants were asked to rate the likability of the program (ranging from 1=“I did not like it at all” to 5=“I liked it a lot”), usefulness (1=“Not useful at all,” 5=“Very useful”), and whether they would recommend the program to other health care workers (1=“Not at all,” 5=“Definitely would recommend”). In the TG, participants were also asked to evaluate the short message prompts they received (0=“Very negatively,” 10=“Very positively”).

#### Use

During the preintervention assessment, participants were asked how actively they expected to use the program, where 1 was “I will not use it” and 10 was “I will use it a lot.” At the postintervention evaluation, participants were asked how actively they actually had used the program (1=“I did not use it” and 10=“I used it a lot”) and how much time on average per week they had managed to devote to the program (1=“Not at all” and 6=“>2 hours”). The congruence of use expectations was measured by subtracting the score reflecting the preintervention expected usage from the postintervention assessment of subjective usage. Moreover, participants’ usage information was exported directly from the platform. Data were collected on the number of logins, modules opened and completed (from 0 to 6), exercises completed (from 0 to 19), messages received from the therapist, and messages sent to the therapist.

### Statistical Analysis

To assess the effect of the program on primary (stress recovery) and secondary outcomes (perceived stress levels, anxiety, depression, and psychological well-being), a Latent Change Modeling (LCM) approach [23] using Mplus 8.8 [24] was carried out. To estimate the within-group effects of the standard intervention and the intervention supplemented by tailored prompts, a series of multigroup LCMs were performed, reporting changes in primary and secondary outcomes from preintervention to postintervention and from preintervention to 6-month follow-up in each group separately. To calculate the between-group effects, a series of conditional LCM was computed in a full sample by regressing the intervention condition (0=SG; 1=TG) on the changes in outcome scores. Moreover, we ran a series of univariate regression analyses to explore whether prompt timing and frequency were associated with changes in outcomes in the TG. A Maximum Likelihood with Robust SE estimator was used in latent change analyses. The Full Information Maximum Likelihood algorithm was used for handling the missing data. Between-group and within-group effect sizes were calculated according to the guidelines for calculating the correct effect size in the LCM [25]. The effect sizes were interpreted according to the recommendations of Cohen (1988) [26], that is 0.20=small effect, 0.50=medium effect, and 0.80=large effect.

In addition, IBM SPSS (version 28) was used to compare the demographic, work-related, and psychological support factors between the TG and the SG using the Student *t* test and chi-square test. Differences between RCT groups and subgroups on program usage and evaluation factors were compared using Student *t* test and ANOVA with the Bonferroni post hoc test.

## Results

### Overview

**Figure 2:**
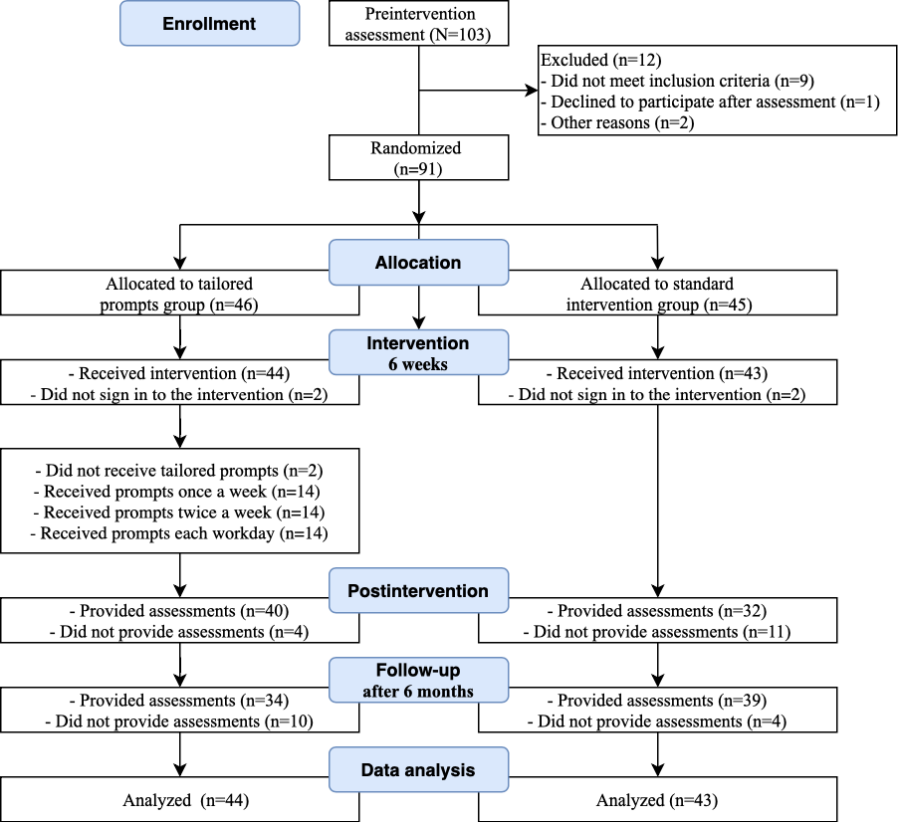
Study flowchart.

The study flowchart is shown in Figure 2. More than 100 individuals registered to participate in the intervention. In total, 91 individuals met eligibility criteria and were randomized to one of the 2 study groups: TG (n=46) or SG (n=45). A total of four participants did not log in to the program (2 from each group) and were therefore not included in the data analysis. The final data analysis included 87 participants (n_TG_=44, n_SG_=43).

### Participant Characteristics

The participants included in the study were almost exclusively female (86/87, 99%). Participants ranged in age from 19 to 68 (mean 39.61, SD 11.49) years. Around a quarter of the participants were licensed medical doctors (24/87, 28%), 9% (8/87) were resident doctors, 31% (27/87) nurses, and 32% (28/87) other health care workers (psychologists, social workers, complementary and alternative health professionals, public health professionals, and oral care professionals). The SG and the TGs did not differ significantly in the preintervention (baseline) measures of sociodemographic, work-related, psychological support, and mental health factors. The sample characteristics of the groups at baseline are shown in Table 2.

**Table 2:** Sample characteristics of standard intervention and tailored prompts groups at baseline.

		Standard intervention group (n=43)	Tailored prompts group (n=44)	Significance statistics	
**Gender,** **n** **(%)**					*χ*^2^_1_=1.0; *P*=.31
	Women	42 (98)	44 (100)		
	Men	1 (2)	0 (0)		
**Age (years)**					*t*_85_=–0.06; *P*=.95
	Mean (SD)	39.53 (11.09)	39.68 (11.99)		
	Range	19-68			
**Education,** **n** **(%)**					*χ*^2^_2_=0.7; *P*=.69
	Secondary or lower	2 (5)	3 (7)		
	Postsecondary or professional college	10 (23)	13 (30)		
	University	31 (72)	28 (64)		
**Long-term relationship,** **n** **(%)**					*χ*^2^_1_=0.03; *P*=.86
	No	11 (26)	12 (27)		
	Yes	32 (74)	32 (73)		
**Position,** **n (** **%)**					*χ*^2^_3_=1.6; *P*=.67
	Medical doctor	11 (26)	13 (30)		
	Resident doctor	5 (12)	3 (7)		
	Nurse	15 (35)	12 (27)		
	Other	12 (28)	16 (36)		
**Work status,** **n** **(%)**					*χ*^2^_2_=1.8, *P*=.42
	Part-time	2 (5)	5 (11)		
	Full-time	18 (42)	20 (46)		
	> Full-time	23 (54)	19 (43)		
**Type of services** ^a^ **,** **n** **(%)**					
	Outpatient	19 (44)	26 (59)	*χ*^2^_1_=1.9; *P*=.16	
	Inpatient	18 (42)	11 (25)	*χ*^2^_1_=2.8; *P*=.10	
	Rehabilitation	3 (7)	5 (11)	*χ*^2^_1_=0.5; *P*=.48	
	Nursing	9 (21)	4 (9)	*χ*^2^_1_=2.4; *P*=.12	
	Paramedics	9 (21)	5 (11)	*χ*^2^_1_=1.5; *P*=.23	
	Intensive care	1 (2)	5 (11)	*χ*^2^_1_=2.8; *P*=.10	
**Work experience,** **n** **(%)**					*χ*^2^_3_=0.4; *P*=.93
	< 2 years	6 (14)	7 (16)		
	2-5 years	11 (26)	9 (21)		
	6-10 years	3 (7)	4 (9)		
	>10 years	23 (54)	24 (55)		
**In psychological treatment,** **n** **(%)**					*χ*^2^_1_=0.6; *P*=.44
	No	38 (88)	41 (93)		
	Yes	5 (12)	3 (7)		
**Taking medication due to mental health difficulties,** **n** **(%)**					*χ*^2^_1_=1.0; *P*=.32
	No	42 (98)	41 (93)		
	Yes	1 (2)	3 (7)		
**Recent use of other self-help apps,** **n** **(%)**					*χ*^2^_1_=2.97; *P*=.09
	No	41 (95)	37 (84)		
	Yes	2 (5)	7 (16)		
**Mental health at baseline, mean (SD)**					
	Recovery from stress	51.16 (9.23)	51.98 (9.06)	*t*_85_=–0.42; *P*=.68	
	Perceived stress	7.56 (2.33)	7.52 (3.02)	*t*_85_=0.06; *P*=.95	
	Anxiety	2.81 (1.47)	3.16 (1.66)	*t*_85_=–1.03; *P*=.31	
	Depression	2.47 (1.50)	2.71 (1.69)	*t*_85_=–0.70; *P*=.49	
	Psychological well-being	40.84 (15.68)	39.09 (19.23)	*t*_85_=0.46; *P*=.64	

^a^Multiple-answer question.

### Use and Support Received

In the TG (n=44), the majority of individuals included in the study opted to receive SMS prompts (42/44, 96%), with a majority choosing to receive them once a week in the afternoon (10/47, 37%). Only 2 participants (5%) preferred not to receive supplementary prompts. The distribution of message preferences for all participants who took part in the preintervention interviews (n=47) is shown in Table S1 in Multimedia Appendix 2.

All participants included in the study logged in to the program up to 27 (mean 8.69, SD 6.02) times. Student *t* test showed no significant difference in the number of logins when comparing the TG (mean 9.27, SD 5.84) and the SG (mean 8.09, SD 6.22, *t*_85_=–0.91; *P*=.36). More than half of the participants (50/87, 58%) of the full sample opened all program modules; but there was no significant difference in the number of program modules opened between the TG (mean 4.89, SD 1.82) and SG (mean 4.28, SD 2.03; *t*_83.57_=–1.47; *P*=.15). Around one-fifth of participants (18/87, 21%) from the full sample fully completed all 6 program modules (Table 3); as previously, no difference between the TG (mean 3.25, SD 2.15) and SG (mean 2.91, SD 2.38) in completed modules was observed (*t*_85_=–0.71; *P*=.48). There was no statistically significant difference between the TG (mean 12.52, SD 6.73) and SG (mean 10.95, SD 7.21) in relation to the number of program exercises (N=19) completed (*t*_85_=–1.05; *P*=.30).

**Table 3:** Number of program modules completed.

	Total (N=87), n (%)	Standard intervention group (n=43), n (%)	Tailored prompts group (n=44), n (%)
0 modules	17 (20)	10 (23)	7 (16)
1 module	15 (17)	8 (19)	7 (16)
2 modules	3 (3)	3 (7)	0 (0)
3 modules	9 (10)	2 (5)	7 (16)
4 modules	13 (15)	4 (9)	9 (21)
5 modules	12 (14)	7 (16)	5 (11)
6 modules	18 (21)	9 (21)	9 (21)

When asked how much time participants spent using the program on average per week, 33% (24/72) indicated that they spent less than 15 minutes, 40% (29/72) spent 15 to 30 minutes, and 26% (19/72) spent more than an hour. There was no difference between the 2 RCT groups with regard to the average time spent while using the program (*χ*^2^_2_=1.1; *P*=.57).

Participants reported using the program significantly less actively at postintervention (mean 6.33, SD 2.25) than they thought they would at the time of the first measurement (mean 8.25, SD 6.33; *t*_72_=6.63; *P*<.001). No significant difference was found between the 2 groups and the congruence of use expectations (measured by subtracting the score reflecting preintervention expected usage from postintervention assessment of usage; Mean_SG_ –1.91, SD_SG_ 2.49; n_SG_ 33; Mean_TG_=–1.93, SD_TG_ 2.49, n_TG_ 40; *t*_71_=0.03, *P*=.98). However, ANOVA analysis (Table S2 in Multimedia Appendix 2) showed that, on average, participants in the TG who chose to receive SMS reminders each workday (mean –3.67, SD 1.78) found themselves using the program less actively than they had expected at preintervention assessment in comparison with those who chosen to receive reminders only once a week (mean –0.39, SD 2.29; mean difference=3.28, *P*=.003, 95% CI 0.92-5.64).

In total, program users received from 0 to 8 messages from their therapists. There was no significant difference between therapist support messages received in the TG (mean 4.36, SD 1.93) and the SG (mean 3.98, SD 2.10; *t*_85_=–0.90, *P*=.37). Participants sent 0 to 5 messages to their therapists, with the majority (64/87, 74%) not contacting them. One-fifth of the participants (19/87, 22%) sent 1 message to their therapist, and only 4/87 (5%) contacted the therapist more than once. No difference was found between the TG and the SG in messages sent (*χ*^2^_2_=1.1; *P*=.58).

### Intervention Effects

**Figure 3:**
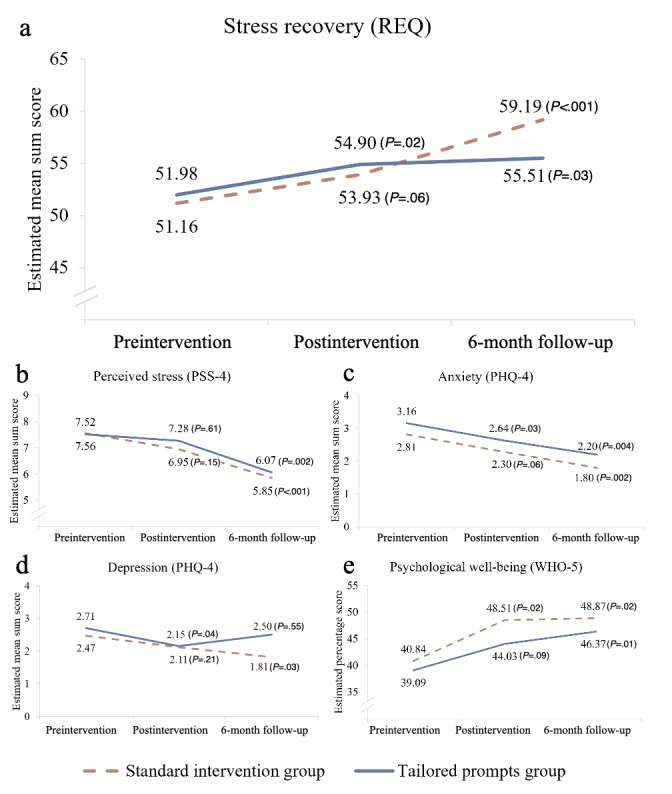
Trajectories of changes in primary and secondary outcomes in the groups of participants receiving standard intervention (n=43) and intervention supplemented with tailored SMS prompts (n=44). Significant statistics are presented for the within-group outcomes from baseline to postintervention and from baseline to 6-month follow-up. PHQ-4: Patient Health Questionnaire-4; PSS-4: Perceived Stress Scale-4; REQ: Recovery Experiences Questionnaire; WHO-5: World Health Organization-5.

The trajectories of outcomes in each group are shown in Figure 3, and Table 4 presents outcome means and SD. Within-group effect sizes and 95% CI are presented in Table 5. There was a significant change in the TG (*P*=.02) and a nonsignificant change in the level of stress recovery in the SG (*P*=.06) at the postintervention assessment (Figure 3, graph A). The effects of the change from preintervention to postintervention were small for both groups. The results were significant at the 6-month follow-up, with small effects in the TG (*P*=.03) and large effects in the SG (*P*<.001). In both groups, however, there were no significant changes in perceived stress levels at postintervention assessment (TG *P*=.61, SG *P*=.15). A significant decrease in perceived stress levels was observed at the 6-month follow-up, with moderate effects in the SG (*P*<.001) and moderate effects in the TG (*P*=.002; Table 5).

Changes in anxiety, depression, and psychological well-being in both groups were also tested (Table 5). The analysis showed a significant (*P*=.03) reduction in anxiety in the TG and a nonsignificant (*P*=.06) reduction in the SG at the postintervention assessment, with small effects. In both groups, this change was significant (*P*<.01) after 6 months, with moderate effects. In addition, depression levels decreased significantly (*P*=.04) in the TG after the intervention but were no longer significant (*P*=.55) at 6-month follow-up. In contrast, in the SG, the level of depression was significantly (*P*=.03) reduced at the 6-month follow-up but not at the postintervention (*P*=.21). The intervention effects on depression were small for both groups (Table 5). Psychological well-being after the intervention increased significantly (*P*=.02) in the SG but not in the TG (*P*=.09). Although, at 6-month follow-up, the increase compared with baseline was significant in both groups (*P*<.05), with small to moderate effects.

When comparing the TG with the SG, there was no significant difference in the level of stress recovery immediately after the intervention (β=0.01, *P*=.93). However, there was a significant difference between the groups when comparing the changes 6 months after the intervention (β=–0.24, *P*=.03), indicating that the group with the standard intervention had a greater increase in recovery from stress after 6 months as compared with the group receiving tailored prompts, with a moderate between-group effect (Table 5). As for secondary outcomes, there was no significant difference in the changes of stress, anxiety, depression, and psychological well-being after the intervention when comparing the TG with the SG, nor was there a significant difference in changes 6 months after the intervention. In the TG, there was no association between changes in outcomes after the intervention and 6-month follow-up and the frequency and timing of received prompts. However, a significant positive association was found between perceived stress levels at baseline and choosing to receive more frequent prompts (β=0.27, *P*=.03) and to receive them in the afternoon (β=0.42, *P*<.001). The full results of multiple univariate regression analyses of the intervention outcomes are presented in Table S3 in Multimedia Appendix 2.

**Table 4:** Means and SD of study outcomes at preintervention, postintervention, and 6 months follow-up.

	Standard intervention group (n=43)				Tailored prompts group (n=44)		
	T1^a^, mean (SD)	T2^b^, mean (SD)	T3^c^, mean (SD)	T1, mean (SD)		T2, mean (SD)	T3, mean (SD)
Stress recovery	51.16 (9.12)	53.93 (8.50)	59.19 (9.54)	51.98 (8.96)		54.90 (7.81)	55.51 (9.49)
Perceived stress	7.56 (2.31)	7.28 (2.41)	6.07 (2.51)	7.52 (2.50)		7.28 (3.11)	6.07 (3.56)
Anxiety	2.81 (1.45)	2.30 (1.62)	1.80 (1.65)	3.16 (1.64)		2.64 (1.61)	2.20 (1.69)
Depression	2.47 (1.48)	2.11 (1.61)	1.81 (1.90)	2.71 (1.67)		2.15 (1.67)	2.50 (2.04)
Psychological well-being	40.84 (15.49)	48.51 (17.45)	48.87 (19.31)	39.09 (19.01)		44.03 (18.00)	46.37 (17.40)

^a^T1: preintervention.

^b^T2: postintervention.

^c^T3: 6-month follow-up.

**Table 5:** Intervention effect sizes.

Group		Within-group effect size		Between-group effect size			
		Pre-post *d*^a^ (95% CI)	Pre-follow-up *d* (95% CI)	Pre-post *d* (95% CI)		Pre-follow-up *d* (95% CI)	
**Stress recovery**					0.02 (–0.40 to 0.44)		–0.49 (–0.92 to –0.07)
	SG^b^	0.31 (–0.11 to 0.74)	0.85 (0.41 to 1.29)				
	TG^c^	0.34 (–0.08 to 0.77)	0.38 (–0.04 to 0.80)				
**Perceived stress**					0.15 (–0.27 to 0.57)		0.10 (–0.32 to 0.52)
	SG	–0.26 (–0.68 to 0.17)	–0.70 (–1.14 to –0.27)				
	TG	–0.08 (–0.50 to 0.33)	–0.47 (–0.89- to 0.04)				
**Anxiety**					0.00 (–0.42 to 0.42)		0.04 (–0.38 to 0.46)
	SG	–0.33 (–0.75 to 0.10)	–0.64 (–1.08 to –0.21)				
	TG	–0.32 (–0.74 to 0.10)	–0.57 (–1.00 to –0.15)				
**Depression**					–0.13 (–0.55 to 0.29)		0.28 (–0.14 to 0.71)
	SG	–0.23 (–0.65 to 0.19)	–0.38 (–0.81 to 0.04)				
	TG	–0.33 [–0.75 to 0.09)	–0.11 [–0.53 to 0.31)				
**Psychological well-being**					–0.16 (–0.58 to 0.26)		–0.04 (–0.46 to 0.38)
	SG	0.46 (0.03 to 0.89)	0.45 (0.03 to 0.88)				
	TG	0.26 (–0.16 to 0.68)	0.40 (–0.03 to 0.82)				

^a^*d*: Cohen *d*.

^a^SG: standard intervention group.

^b^TG: tailored prompts group.

### Acceptability

In the TG (n=44), out of those who provided a rating (n=40), most participants (39/40, 98%) rated prompting positively (5< on a scale from 0 to 10), and only 1 person (2%) did not like the prompts received (≤5). Moreover, most of the participants who provided a rating found the program to be useful (58/72, 81%) regardless of the RCT group (*χ*^2^_4_=3.6; *P*=.46). There was no significant group effect with regard to participants liking the program (*χ*^2^_4_=2.8; *P*=.60), with 88% (63/72) in the total sample indicating that they overall liked it. Most of the participants (66/72, 92%) indicated that they would recommend the program to other health care workers regardless of the group (*χ*^2^_4_=2.8; *P*=.60).

## Discussion

### Principal Results

The aim of this study was to test whether the inclusion of tailored prompts to pursue the individual usage intensity goal would increase engagement and efficacy of ICBT stress recovery intervention for health care workers. While users expressed satisfaction with the intervention and received supplementary prompts, results revealed that tailored prompts had no significant effects on usage indicators and were not associated with additional stress recovery, perceived stress, anxiety, depression, or psychological well-being outcomes. The results thus bring new insights to the field of research on internet interventions and call to consider the possible effects of supplementary tailored prompts when designing or testing internet interventions.

### Comparisons With Previous Work

This study was the first to support that the effects of an internet-delivered stress recovery intervention are maintained after 6 months. In contrast to previous trials, reporting positive mental health effects after 3 months [4,5], participants in the current study exhibited significant improvements when using the standard intervention in primary (stress recovery) and secondary outcomes (stress, anxiety, depression, and psychological well-being) 6 months postintervention, with a small to large effect size. However, in the intervention supplemented by tailored prompts, the decrease in depression was no longer significant at the 6-month follow-up, and the effects on stress recovery, stress, anxiety, and psychological well-being were small to moderate despite the overall positive evaluation of tailored SMS prompts. These findings affirm the efficacy of the internet-delivered stress recovery intervention in fostering enduring improvements in the well-being of health care workers, even without additional administrative resources to enhance engagement. Incorporating an optional therapist support program format [5] could further reduce costs, facilitating the scalability of the intervention to a national level.

We did not observe significant differences between the RCT groups in terms of the program’s effects on stress, anxiety, depression, or psychological well-being. However, tailored prompts were associated with a smaller, albeit significantly improved, intervention effect on the primary outcome: stress recovery. A possible explanation for this result may refer to control and mastery, key elements of stress recovery, which include choosing leisure activities, deciding when and how to engage in them, and experiencing a sense of competence and self-efficacy [15]. It is possible that constant reminders to take time to unwind could inhibit the development of these skills. Moreover, the unanticipated effect of tailored prompts may refer to the thwarted sense of agency, that is, attributing improvement to oneself rather than to others, for example, researchers, which is positively related to the effectiveness of therapy [27]. Participants who did not receive tailored prompts were able to attribute success to themselves, which may have led to more developed stress-coping skills. In addition, tailored reminders to make time for the program according to the goals set act as self-monitoring, which can have negative effects, for instance, when feeling guilty about not achieving goals [13]. Finally, previous studies have also found that external motivation, that is feeling pressured to complete tasks, can sometimes be counterproductive and lead to smaller treatment gains [28]. These findings highlight the importance of designing prompts that encourage engagement while preserving participants’ sense of autonomy, as this is crucial for effective change [29].

The trial did not find that tailored SMS prompts positively impacted health care workers’ engagement in an internet-delivered stress recovery intervention. To begin with, we found no differences in the program use indicators (eg, number of logins and exercises completed) between the 2 study groups. Similarly, a study reported that adaptive tailoring of notification timing does not enhance the use of a smartphone-based stress management app [12]. On the other hand, we found that participants who received tailored prompts were more likely to use the program more than they expected before the intervention. However, this may also reflect a perception that the program required more of their time than they would have intended. It is also likely that SMS prompts may have an impact on other adherence variables not measured in this study, such as faster login and login duration [30]. However, we should bear in mind that while adherence is important, involvement is possibly a stronger predictor of intervention effects, and it has been suggested that it may act as a working mechanism for persuasive technologies [8]. Thus, further research should test other factors of engagement that might be influenced by including tailored prompts and clarify what works for whom to ensure optimal participant engagement.

This study yields new insights into the complex interplay between users’ expectations, goal setting, engagement, tailoring, and prompting in an internet-delivered cognitive behavior therapy for recovery from stress. Tailoring such programs for health care workers poses unique challenges due to their often fluctuating and unpredictable schedules, which can render initially appropriate treatment components less suitable over time. A recent qualitative analysis highlighted that unmet expectations and workload are significant barriers to engagement in ICBT and noted that even tailored prompts to engage can be perceived as unsuitable or stressful [6]. These findings suggest that supplementary prompts, though possibly beneficial for some users (eg, those with more predictable schedules or those less motivated), may not be universally effective. Discontinuation of ICBT can hinge on the client’s expectations, treatment credibility, and intrinsic motivation [28], which can be shaped through techniques such as goal setting [13], motivational interviews [31], or educational videos [32]. Reminders could also support motivation and prompt clients to reach treatment goals [33], but it is essential to allow for the ongoing adjustment of these strategies to align with users’ changing needs and circumstances. Future research should further explore different factors that influence engagement and the efficacy of internet interventions and the mechanisms driving their success. These insights will be crucial for advancing the design and delivery of psychological treatments.

### Limitations

The results of this study should be viewed in the context of its limitations. Even though previous research has found the tested stress recovery intervention to be effective at postintervention assessment [4,5], in the current study, no significant changes in the perceived stress at postintervention were found in comparison with baseline assessment. This could be explained by one of the shortcomings of this study, the modest sample size. As well as different measures used, as the short version of the PSS scale had relatively low reliability in our study, it may not have captured more nuanced changes. Second, the results of this study cannot be generalized to a broader population, as participants were almost exclusively female and health care workers. Finally, the absence of a third control group that did not receive ICBT limits comparison possibilities. Despite the limitations, this novel study provides a further understanding of how internet intervention effects can be influenced by the inclusion of tailored prompts to achieve usage goals.

### Conclusions

The results of this study have highlighted that some techniques for the promotion of engagement in internet interventions, in this case, SMS prompts, may not necessarily have a beneficial effect, even if they are tailored to the needs of participants. Thus, when seeking to improve stress recovery skills in a sample of health care workers, it is important to look for factors other than tailored prompts that determine engagement and treatment success. Finally, the developers of internet-delivered interventions should carefully consider if their intervention should be supplemented by tailored prompts, even if they are broadly acceptable, as they may undermine the acquisition of some skills targeted.

## Appendix

Multimedia Appendix 1 CONSORT-EHEALTH checklist (V 1.6.1).

Multimedia Appendix 2 Additional material.

## Abbreviations

CONSORT-EHEALTH: Consolidated Standards of Reporting Trials of Electronic and Mobile Health Applications and Online Telehealth
FOREST: For Recovery from Stress
HCW: health care workers
ICBT: internet-delivered cognitive behavior therapy
LCM: latent change modeling
PHQ-4: Patient Health Questionnaire-4
PSS-4: Perceived Stress Scale-4
RCT: randomized controlled trial
REQ: Recovery Experiences Questionnaire
SG: standard intervention group
TG: tailored prompts group
WHO-5: World Health Organization-5 Well-Being Index
