# Supplementary material for: Role of Tailored Timing and Frequency Prompts on the Efficacy of an Internet-Delivered Stress Recovery Intervention for Health Care Workers: Randomized Controlled Trial
Source: JMIR Ment Health. 2025 Jan 28;12:e62782. doi: 10.2196/62782 (PMC11815303; doi:10.2196/62782)
Supplement: Multimedia Appendix 2 [file mental_v12i1e62782_app2.docx]

**Multimedia Appendix 1**

Table 1. Preferences on the timing and frequency of prompts at the preintervention interview in the tailored prompts group.

|  | Timing (*n)* | | | |  |
| --- | --- | --- | --- | --- | --- |
| Frequency | AM | AM & PM | PM | Not important | *Total* |
| Twice a day each workday | 0 | 1 | 0 | 0 | 1 |
| Each workday | 4 | 0 | 9 | 1 | 14 |
| Twice a week | 5 | 0 | 9 | 1 | 15 |
| Once a week | 5 | 0 | 10 | 0 | 15 |
| No additional reminders | 0 | 0 | 0 | 2 | 2 |
| *Total* | 14 | 1 | 28 | 4 | 47 |

*Note*. AM – before noon, PM – after noon.

Table 2. Difference in usage expectations and actual usage in different prompting frequency groups.

|  | Congruence of program use expectations | | | | | |
| --- | --- | --- | --- | --- | --- | --- |
| Frequency of prompting | *n* | *M* | *SD* | *F* | *df* | *P* |
| No prompts | 35 | 1.77 | 2.56 | 4.30 | 3 | .008 |
| Once a week | 13 | 0.39 | 2.29 |  |  |  |
| Twice a week | 13 | 2.23 | 2.05 |  |  |  |
| Each workday | 12 | 3.67 | 1.78 |  |  |  |

Table 3. The results of univariate regression analyses of intervention outcomes.

|  |  |  | Regression effects (β coefficients) | | |
| --- | --- | --- | --- | --- | --- |
| Outcome | Predictor | *n* | Baseline | Pre- to postintervention | Preintervention to follow-up |
| Stress recovery | RCT group | 87 | .05 | .01 | **-.24*** |
|  | Frequency (TG) | 44 | -.22 | -.11 | .17 |
|  | Timing (TG) | 42 | -.16 | -.07 | -.08 |
| Perceived stress | RCT group | 87 | -.01 | .07 | .04 |
|  | Frequency (TG) | 44 | **.27*** | -.28 | -.29 |
|  | Timing (TG) | 42 | **.42***** | -.07 | -.12 |
| Anxiety | RCT group | 87 | .11 | -.00 | .02 |
|  | Frequency (TG) | 44 | .10 | -.11 | -.15 |
|  | Timing (TG) | 42 | -.04 | -.05 | .09 |
| Depression | RCT group | 87 | .08 | -.06 | .13 |
|  | Frequency (TG) | 44 | .09 | -.07 | -.04 |
|  | Timing (TG) | 42 | .06 | -.05 | .10 |
| Psychological well-being | RCT group | 87 | -.05 | -.08 | -.05 |
|  | Frequency (TG) | 44 | -.11 | -.08 | -.01 |
|  | Timing (TG) | 42 | -.03 | .20 | .01 |

*Note*. Effects of frequency and timing of prompting are calculated only for the tailored prompts group (TG). * *P* < .05, ** *P* < .01, *** *P* < .001.
