# Supplementary material for: Role of Tailored Timing and Frequency Prompts on the Efficacy of an Internet-Delivered Stress Recovery Intervention for Health Care Workers: Randomized Controlled Trial
Source: JMIR Ment Health. 2025 Jan 28;12:e62782. doi: 10.2196/62782 (PMC11815303; doi:10.2196/62782)
Supplement: Multimedia Appendix 1 [file mental_v12i1e62782_app1.pdf]

CONSORT-EHEALTH (V 1.6.1) - Submission/Publication Form

The CONSORT-EHEALTH checklist is intended for authors of randomized trials evaluating web-based and Internet-based applications/interventions, including mobile interventions, electronic games (incl multiplayer games), social media, certain telehealth applications, and other interactive and/or networked electronic applications. Some of the items (e.g. all subitems under item 5 - description of the intervention) may also be applicable for other study designs.

The goal of the CONSORT EHEALTH checklist and guideline is to be
a) a guide for reporting for authors of RCTs,
b) to form a basis for appraisal of an ehealth trial (in terms of validity)

CONSORT-EHEALTH items/subitems are MANDATORY reporting items for studies published in the Journal of Medical Internet Research and other journals / scientific societies endorsing the checklist.

Items numbered 1., 2., 3., 4a., 4b etc are original CONSORT or CONSORT-NPT (non-pharmacologic treatment) items.
Items with Roman numerals (i., ii, iii, iv etc.) are CONSORT-EHEALTH extensions/clarifications.

As the CONSORT-EHEALTH checklist is still considered in a formative stage, we would ask that you also RATE ON A SCALE OF 1-5 how important/useful you feel each item is FOR THE PURPOSE OF THE CHECKLIST and reporting guideline (optional).

Mandatory reporting items are marked with a red *.
In the textboxes, either copy & paste the relevant sections from your manuscript into this form - please include any quotes from your manuscript in QUOTATION MARKS,
or answer directly by providing additional information not in the manuscript, or elaborating on why the item was not relevant for this study.

YOUR ANSWERS WILL BE PUBLISHED AS A SUPPLEMENTARY FILE TO YOUR PUBLICATION IN JMIR AND ARE CONSIDERED PART OF YOUR PUBLICATION (IF ACCEPTED).
Please fill in these questions diligently. Information will not be copyedited, so please use proper spelling and grammar, use correct capitalization, and avoid abbreviations.

DO NOT FORGET TO SAVE AS PDF _AND_ CLICK THE SUBMIT BUTTON SO YOUR ANSWERS ARE IN OUR DATABASE !!!

Citation Suggestion (if you append the pdf as Appendix we suggest to cite this paper in the caption):
Eysenbach G, CONSORT-EHEALTH Group
CONSORT-EHEALTH: Improving and Standardizing Evaluation Reports of Web-based and Mobile Health Interventions
J Med Internet Res 2011;13(4):e126
URL: http://www.jmir.org/2011/4/e126/
doi: 10.2196/jmir.1923
PMID: 22209829

auguste.nomeikaite@gmail.com
Perjungti paskyrą
Pateikite iš naujo, kad išsaugotumėte
Nebendrinamas

* Nurodo būtiną klausimą

Your name *
First Last

Auguste Nomeikaite

Primary Affiliation (short), City, Country *
University of Toronto, Toronto, Canada

Vilnius university, Vilnius, Lithuania

Your e-mail address *
abc@gmail.com

auguste.nomeikaite@fsf.vu.lt

Title of your manuscript *
Provide the (draft) title of your manuscript.

The Role of Tailored Timing and Frequency Prompts on the Efficacy of an Internet-Delivered Stress Recovery Intervention for Healthcare Workers: A Randomized Controlled Trial

Name of your App/Software/Intervention *
If there is a short and a long/alternate name, write the short name first and add the long name in brackets.

FOREST+ (For Recovery from Stress)

Evaluated Version (if any)
e.g. "V1", "Release 2017-03-01", "Version 2.0.27913"

Version: V2

Language(s) *
What language is the intervention/app in? If multiple languages are available, separate by comma (e.g. "English, French")

Lithuanian

URL of your Intervention Website or App
e.g. a direct link to the mobile app on app in appstore (itunes, Google Play), or URL of the website. If the intervention is a DVD or hardware, you can also link to an Amazon page.

www.forestmedikams.lt

URL of an image/screenshot (optional)

Jūsų atsakymas

Accessibility *
Can an enduser access the intervention presently?

- ( ) access is free and open
- ( ) access only for special usergroups, not open
- ( ) access is open to everyone, but requires payment/subscription/in-app purchases
- (•) app/intervention no longer accessible
- ( ) Kita:

Primary Medical Indication/Disease/Condition *
e.g. "Stress", "Diabetes", or define the target group in brackets after the condition, e.g. "Autism (Parents of children with)", "Alzheimers (Informal Caregivers of)"

Sress recovery (Healthcare staff)

Primary Outcomes measured in trial *
comma-separated list of primary outcomes reported in the trial

Recovery Experiences Questionnaire (REQ)

Secondary/other outcomes
Are there any other outcomes the intervention is expected to affect?

Perceived Stress Scale (PSS-4), Patient Health Questionnaire (PHQ-4), and World Health Organization-Five Well-Being Index (WHO-5)

Recommended "Dose" *
What do the instructions for users say on how often the app should be used?

- ( ) Approximately Daily
- ( ) Approximately Weekly
- ( ) Approximately Monthly
- ( ) Approximately Yearly
- ( ) "as needed"
- (•) Kita: Access to new module provided weekly, but daily use encouraged

Approx. Percentage of Users (starters) still using the app as recommended after 3 months *

- ( ) unknown / not evaluated
- ( ) 0-10%
- ( ) 11-20%
- ( ) 21-30%
- ( ) 31-40%
- ( ) 41-50%
- ( ) 51-60%
- ( ) 61-70%
- ( ) 71%-80%
- ( ) 81-90%
- ( ) 91-100%
- (•) Kita: Access was limited to 2 months

Overall, was the app/intervention effective? *

- (•) yes: all primary outcomes were significantly better in intervention group vs control
- ( ) partly: SOME primary outcomes were significantly better in intervention group vs control
- ( ) no statistically significant difference between control and intervention
- ( ) potentially harmful: control was significantly better than intervention in one or more outcomes
- ( ) inconclusive: more research is needed
- ( ) Kita:

Article Preparation Status/Stage *
At which stage in your article preparation are you currently (at the time you fill in this form)

- ( ) not submitted yet - in early draft status
- ( ) not submitted yet - in late draft status, just before submission
- ( ) submitted to a journal but not reviewed yet
- (•) submitted to a journal and after receiving initial reviewer comments
- ( ) submitted to a journal and accepted, but not published yet
- ( ) published
- ( ) Kita:

Journal *
If you already know where you will submit this paper (or if it is already submitted), please provide the journal name (if it is not JMIR, provide the journal name under "other")

- ( ) not submitted yet / unclear where I will submit this
- ( ) Journal of Medical Internet Research (JMIR)
- ( ) JMIR mHealth and UHealth
- ( ) JMIR Serious Games
- (•) JMIR Mental Health
- ( ) JMIR Public Health
- ( ) JMIR Formative Research
- ( ) Other JMIR sister journal
- ( ) Kita:

Is this a full powered effectiveness trial or a pilot/feasibility trial? *

- ( ) Pilot/feasibility
- (•) Fully powered

Manuscript tracking number *
If this is a JMIR submission, please provide the manuscript tracking number under "other" (The ms tracking number can be found in the submission acknowledgement email, or when you login as author in JMIR. If the paper is already published in JMIR, then the ms tracking number is the four-digit number at the end of the DOI, to be found at the bottom of each published article in JMIR)

- ( ) no ms number (yet) / not (yet) submitted to / published in JMIR
- (•) Kita: ms #62782

TITLE AND ABSTRACT

1a) TITLE: Identification as a randomized trial in the title

1a) Does your paper address CONSORT item 1a? *
I.e does the title contain the phrase "Randomized Controlled Trial"? (if not, explain the reason under "other")

- (•) yes
- ( ) Kita:

1a-i) Identify the mode of delivery in the title
Identify the mode of delivery. Preferably use "web-based" and/or "mobile" and/or "electronic game" in the title. Avoid ambiguous terms like "online", "virtual", "interactive". Use "Internet-based" only if Intervention includes non-web-based Internet components (e.g. email), use "computer-based" or "electronic" only if offline products are used. Use "virtual" only in the context of "virtual reality" (3-D worlds). Use "online" only in the context of "online support groups". Complement or substitute product names with broader terms for the class of products (such as "mobile" or "smart phone" instead of "iphone"), especially if the application runs on different platforms.

subitem not at all important ( ) 1 ( ) 2 ( ) 3 ( ) 4 ( ) 5 essential

Does your paper address subitem 1a-i? *
Copy and paste relevant sections from manuscript title (include quotes in quotation marks "like this" to indicate direct quotes from your manuscript), or elaborate on this item by providing additional information not in the ms, or briefly explain why the item is not applicable/relevant for your study

"Internet-Delivered" is included in the title

1a-ii) Non-web-based components or important co-interventions in title
Mention non-web-based components or important co-interventions in title, if any (e.g., "with telephone support").

subitem not at all important ( ) 1 ( ) 2 ( ) 3 ( ) 4 ( ) 5 essential

Does your paper address subitem 1a-ii?
Copy and paste relevant sections from manuscript title (include quotes in quotation marks "like this" to indicate direct quotes from your manuscript), or elaborate on this item by providing additional information not in the ms, or briefly explain why the item is not applicable/relevant for your study

"Tailored Timing and Frequency Prompts" is included in the title

1a-iii) Primary condition or target group in the title
Mention primary condition or target group in the title, if any (e.g., "for children with Type I Diabetes") Example: A Web-based and Mobile Intervention with Telephone Support for Children with Type I Diabetes: Randomized Controlled Trial

subitem not at all important ( ) 1 ( ) 2 ( ) 3 ( ) 4 ( ) 5 essential

Does your paper address subitem 1a-iii? *
Copy and paste relevant sections from manuscript title (include quotes in quotation marks "like this" to indicate direct quotes from your manuscript), or elaborate on this item by providing additional information not in the ms, or briefly explain why the item is not applicable/relevant for your study

In the title, we state: "for Healthcare Workers".

1b) ABSTRACT: Structured summary of trial design, methods, results, and conclusions
NPT extension: Description of experimental treatment, comparator, care providers, centers, and blinding status.

1b-i) Key features/functionalities/components of the intervention and comparator in the METHODS section of the ABSTRACT
Mention key features/functionalities/components of the intervention and comparator in the abstract. If possible, also mention theories and principles used for designing the site. Keep in mind the needs of systematic reviewers and indexers by including important synonyms. (Note: Only report in the abstract what the main paper is reporting. If this information is missing from the main body of text, consider adding it)

subitem not at all important ( ) 1 ( ) 2 ( ) 3 ( ) 4 ( ) 5 essential

Does your paper address subitem 1b-i? *
Copy and paste relevant sections from the manuscript abstract (include quotes in quotation marks "like this" to indicate direct quotes from your manuscript), or elaborate on this item by providing additional information not in the ms, or briefly explain why the item is not applicable/relevant for your study

We state: "The aim of this study was to test if prompts tailored based on timing and frequency, aligned with pre-intervention goal setting, can increase usage and the efficacy of a therapist-supported ICBT stress recovery intervention for healthcare workers." and "The two-arm randomized controlled trial included 87 healthcare workers (99% female, aged 19-68 years: M=39.61, SD=11.49): 43 in the standard intervention group and 44 in the tailored prompts group."

1b-ii) Level of human involvement in the METHODS section of the ABSTRACT
Clarify the level of human involvement in the abstract, e.g., use phrases like "fully automated" vs. "therapist/nurse/care provider/physician-assisted" (mention number and expertise of providers involved, if any). (Note: Only report in the abstract what the main paper is reporting. If this information is missing from the main body of text, consider adding it)

subitem not at all important ( ) 1 ( ) 2 ( ) 3 ( ) 4 ( ) 5 essential

Does your paper address subitem 1b-ii?
Copy and paste relevant sections from the manuscript abstract (include quotes in quotation marks "like this" to indicate direct quotes from your manuscript), or elaborate on this item by providing additional information not in the ms, or briefly explain why the item is not applicable/relevant for your study

We state: "The aim of this study was to test if prompts tailored based on timing and frequency, aligned with pre-intervention goal setting, can increase usage and the efficacy of a therapist-supported ICBT stress recovery intervention for healthcare workers."

1b-iii) Open vs. closed, web-based (self-assessment) vs. face-to-face assessments in the METHODS section of the ABSTRACT
Mention how participants were recruited (online vs. offline), e.g., from an open access website or from a clinic or a closed online user group (closed usergroup trial), and clarify if this was a purely web-based trial, or there were face-to-face components (as part of the intervention or for assessment). Clearly say if outcomes were self-assessed through questionnaires (as common in web-based trials). Note: In traditional offline trials, an open trial (open-label trial) is a type of clinical trial in which both the researchers and participants know which treatment is being administered. To avoid confusion, use "blinded" or "unblinded" to indicated the level of blinding instead of "open", as "open" in web-based trials usually refers to "open access" (i.e. participants can self-enrol). (Note: Only report in the abstract what the main paper is reporting. If this information is missing from the main body of text, consider adding it)

subitem not at all important ( ) 1 ( ) 2 ( ) 3 ( ) 4 ( ) 5 essential

Does your paper address subitem 1b-iii?
Copy and paste relevant sections from the manuscript abstract (include quotes in quotation marks "like this" to indicate direct quotes from your manuscript), or elaborate on this item by providing additional information not in the ms, or briefly explain why the item is not applicable/relevant for your study

We state: "The self-report data were collected online at pre-intervention (September 2022), post-intervention (October 2022), and 6-month follow-up (May 2023)."

1b-iv) RESULTS section in abstract must contain use data
Report number of participants enrolled/assessed in each group, the use/uptake of the intervention (e.g., attrition/adherence metrics, use over time, number of logins etc.), in addition to primary/secondary outcomes. (Note: Only report in the abstract what the main paper is reporting. If this information is missing from the main body of text, consider adding it)

subitem not at all important ( ) 1 ( ) 2 ( ) 3 ( ) 4 ( ) 5 essential

Does your paper address subitem 1b-iv?
Copy and paste relevant sections from the manuscript abstract (include quotes in quotation marks "like this" to indicate direct quotes from your manuscript), or elaborate on this item by providing additional information not in the ms, or briefly explain why the item is not applicable/relevant for your study

We state: "The two-arm randomized controlled trial included 87 healthcare workers (99% female, aged 19-68 years: M=39.61, SD=11.49): 43 in the standard intervention group and 44 in the tailored prompts group." and "The results showed that tailored prompts, although appreciated by the majority (98%; n=39), did not improve intervention usage indicators, such as the number of logins (t(85)=-0.91; P=.36), modules opened (t(83.57)=-1.47; P=.15), modules completed (t(85)=-0.71; P=.48), exercises completed (t(85)=-1.05; P=.30), or the time spent using the program (χ2(2)=1.13; P=.57). Similarly, tailored prompts did not increase the effects of the intervention in terms of stress recovery skills (Cohens d ranging from 0.31 to 0.85), perceived stress (d: -0.08; -0.70), depression (d: -0.11; -0.38), anxiety (d: -0.32; -0.64) or psychological well-being (d: 0.26; 0.46). In addition, the standard intervention group showed greater long-term stress recovery effects than the group using the internet-delivered intervention supplemented by tailored prompts (β=-0.24, P=.03)."

1b-v) CONCLUSIONS/DISCUSSION in abstract for negative trials
Conclusions/Discussions in abstract for negative trials: Discuss the primary outcome - if the trial is negative (primary outcome not changed), and the intervention was not used, discuss whether negative results are attributable to lack of uptake and discuss reasons. (Note: Only report in the abstract what the main paper is reporting. If this information is missing from the main body of text, consider adding it)

subitem not at all important ( ) 1 ( ) 2 ( ) 3 ( ) 4 ( ) 5 essential

Does your paper address subitem 1b-v?
Copy and paste relevant sections from the manuscript abstract (include quotes in quotation marks "like this" to indicate direct quotes from your manuscript), or elaborate on this item by providing additional information not in the ms, or briefly explain why the item is not applicable/relevant for your study

Jūsų atsakymas

INTRODUCTION

2a) In INTRODUCTION: Scientific background and explanation of rationale

2a-i) Problem and the type of system/solution
Describe the problem and the type of system/solution that is object of the study: intended as stand-alone intervention vs. incorporated in broader health care program? Intended for a particular patient population? Goals of the intervention, e.g., being more cost-effective to other interventions, replace or complement other solutions? (Note: Details about the intervention are provided in "Methods" under 5)

subitem not at all important ( ) 1 ( ) 2 ( ) 3 ( ) 4 ( ) 5 essential

Does your paper address subitem 2a-i? *
Copy and paste relevant sections from the manuscript (include quotes in quotation marks "like this" to indicate direct quotes from your manuscript), or elaborate on this item by providing additional information not in the ms, or briefly explain why the item is not applicable/relevant for your study

We state: "Healthcare workers (HCWs) are at risk of stress, burnout, and other mental health problems [1,2]. However, long working hours, night shifts, rigid schedules, and prevailing stigma can make it difficult for them to engage in traditional psychological treatment [3]. ICBT could be a viable alternative and has shown efficacy in helping HCWs develop stress recovery skills, reduce stress, anxiety, and depression, and improve overall psychological

well-being [4,5]. However, findings in these studies also indicated that only half of the included participants familiarized themselves with the full content of the intervention. A qualitative study of early dropouts revealed that many identified barriers to engagement, such as a lack of time or motivation and unmet expectations or needs [6]. Across a range of diagnoses, better adherence has emerged as a predictor of better outcomes in internet interventions for adults [7]. Thus, it is crucial to find ways to motivate HCWs' engagement in internet interventions but in a way that considers individual needs and time constraints." and "In this study, we aimed to test whether the inclusion of tailored prompts aimed at achieving usage goals can increase the efficacy of an ICBT stress recovery intervention for healthcare workers in a randomized controlled trial (RCT)."

2a-ii) Scientific background, rationale: What is known about the (type of) system

Scientific background, rationale: What is known about the (type of) system that is the object of the study (be sure to discuss the use of similar systems for other conditions/diagnoses, if appropiate), motivation for the study, i.e. what are the reasons for and what is the context for this specific study, from which stakeholder viewpoint is the study performed, potential impact of findings [2]. Briefly justify the choice of the comparator.

1 2 3 4 5

subitem not at all important ○ ○ ○ ○ ○ essential

Does your paper address subitem 2a-ii? *

Copy and paste relevant sections from the manuscript (include quotes in quotation marks "like this" to indicate direct quotes from your manuscript), or elaborate on this item by providing additional information not in the ms, or briefly explain why the item is not applicable/relevant for your study

We state: "To enhance retention in ICBT, various persuasion techniques, such as text message reminders, have been proposed [8]. The inclusion of prompts to encourage engagement in internet interventions for healthy behavior and mental health has shown promising results [9]. Research findings are, however, inconsistent, with studies finding no significant clinical benefits of supplementary prompts in digital treatment [10]. Indeed, if prompts received are not relevant for the user, they can have the opposite effect and be more obtrusive rather than helpful [11]. Tailoring the frequency and timing of prompts could be a potential solution in internet-delivered stress management treatment [12], although there is still scarce knowledge of whether this affects engagement and intervention effects. Another suggested solution to reducing negative emotions caused by reminders is to set goals [13], such as how much time a user intends to spend on treatment. To conclude, setting usage goals and delivering tailored prompts could be a way to promote the engagement of healthcare workers and consequently increase the efficacy of the internet-delivered stress recovery intervention."

2b) In INTRODUCTION: Specific objectives or hypotheses

Does your paper address CONSORT subitem 2b? *

Copy and paste relevant sections from the manuscript (include quotes in quotation marks "like this" to indicate direct quotes from your manuscript), or elaborate on this item by providing additional information not in the ms, or briefly explain why the item is not applicable/relevant for your study

We state: "In this study, we aimed to test whether the inclusion of tailored prompts aimed at achieving usage goals can increase the efficacy of an ICBT stress recovery intervention for healthcare workers in a randomized controlled trial (RCT). The “For Recovery from Stress” (FOREST) is a 6-week ICBT intervention [14], incorporating mindfulness and focused on stress recovery [15]. FOREST+ is the updated version of FOREST, designed to meet the needs of healthcare workers [5]. Four objectives were set: (1) to analyze associations between tailored prompts and different engagement indicators in a stress recovery intervention; (2) to assess whether the intervention with tailored prompts is more effective in improving stress recovery skills as compared to the standard intervention; (3) to test if the inclusion of tailored prompts can improve the effects of the internet intervention on stress, anxiety, depression, and psychological well-being, and (4) to explore how having an option to receive tailored prompts alters the users program evaluation."

METHODS

3a) Description of trial design (such as parallel, factorial) including allocation ratio

Does your paper address CONSORT subitem 3a? *

Copy and paste relevant sections from the manuscript (include quotes in quotation marks "like this" to indicate direct quotes from your manuscript), or elaborate on this item by providing additional information not in the ms, or briefly explain why the item is not applicable/relevant for your study

We state: "A two-arm randomized controlled trial (RCT) was conducted to investigate how usage goal setting with prompts tailored by timing and frequency would be related to the usage and the efficacy of an internet-delivered intervention for stress recovery. Eligible participants were randomized (1:1) by an independent researcher into two study groups: a standard intervention group or a tailored prompts group, using built-in randomization functionality in the hosting platform."

3b) Important changes to methods after trial commencement (such as eligibility criteria), with reasons

Does your paper address CONSORT subitem 3b? *

Copy and paste relevant sections from the manuscript (include quotes in quotation marks "like this" to indicate direct quotes from your manuscript), or elaborate on this item by providing additional information not in the ms, or briefly explain why the item is not applicable/relevant for your study

No important changes to methods after trial commencement were done.

3b-i) Bug fixes, Downtimes, Content Changes

Bug fixes, Downtimes, Content Changes: ehealth systems are often dynamic systems. A description of changes to methods therefore also includes important changes made on the intervention or comparator during the trial (e.g., major bug fixes or changes in the functionality or content) (5-iii) and other “unexpected events” that may have influenced study design such as staff changes, system failures/downtimes, etc. [2].

1 2 3 4 5

subitem not at all important ○ ○ ○ ○ ○ essential

Does your paper address subitem 3b-i?

Copy and paste relevant sections from the manuscript (include quotes in quotation marks "like this" to indicate direct quotes from your manuscript), or elaborate on this item by providing additional information not in the ms, or briefly explain why the item is not applicable/relevant for your study

No bug fixes, downtimes, content changes were made.

4a) Eligibility criteria for participants

Does your paper address CONSORT subitem 4a? *

Copy and paste relevant sections from the manuscript (include quotes in quotation marks "like this" to indicate direct quotes from your manuscript), or elaborate on this item by providing additional information not in the ms, or briefly explain why the item is not applicable/relevant for your study

We state: "After completing the registration form and the pre-intervention questionnaire, participants were contacted for a brief phone interview to ensure that they met the eligibility criteria for the study: (1) currently working in a health care facility; (2) adult (>18 years); (3) comprehend Lithuanian; (4) access to a device with internet connection. Certain exclusion criteria have also been identified: (1) high risk of suicide; (2) acute psychiatric crisis; (3) exposure to the current interpersonal violence. Participants who did not meet the eligibility criteria (n=9) were referred elsewhere for psychological support if needed."

4a-i) Computer / Internet literacy

Computer / Internet literacy is often an implicit “de facto” eligibility criterion - this should be explicitly clarified.

1 2 3 4 5

subitem not at all important ○ ○ ○ ○ ○ essential

Does your paper address subitem 4a-i?

Copy and paste relevant sections from the manuscript (include quotes in quotation marks "like this" to indicate direct quotes from your manuscript), or elaborate on this item by providing additional information not in the ms, or briefly explain why the item is not applicable/relevant for your study

Jūsų atsakymas

4a-ii) Open vs. closed, web-based vs. face-to-face assessments:

Open vs. closed, web-based vs. face-to-face assessments: Mention how participants were recruited (online vs. offline), e.g., from an open access website or from a clinic, and clarify if this was a purely web-based trial, or there were face-to-face components (as part of the intervention or for assessment), i.e., to what degree got the study team to know the participant. In online-only trials, clarify if participants were quasi-anonymous and whether having multiple identities was possible or whether technical or logistical measures (e.g., cookies, email confirmation, phone calls) were used to detect/prevent these.

1 2 3 4 5

subitem not at all important ○ ○ ○ ○ ○ essential

Does your paper address subitem 4a-ii? *

Copy and paste relevant sections from the manuscript (include quotes in quotation marks "like this" to indicate direct quotes from your manuscript), or elaborate on this item by providing additional information not in the ms, or briefly explain why the item is not applicable/relevant for your study

We state: "The assessments took place on three occasions: (1) pre-intervention (September 2022), (2) post-intervention (October 2022), and (3) at the 6-month follow-up (May 2023). Informed consent was obtained online at the time of enrolment prior to the pre-intervention assessment. Participants' self-reported data and data on the use of the program were collected using a secure platform, Iterapi, which hosted the program [16]." and "The call for participation in the intervention was disseminated nationwide in Lithuania through various social networks, emails to regional and national professional HCWs societies and groups, and healthcare institutions. Those interested could register for the intervention via the intervention website, which provided detailed information about the program and the research study."

4a-iii) Information giving during recruitment

Information given during recruitment. Specify how participants were briefed for recruitment and in the informed consent procedures (e.g., publish the informed consent documentation as appendix, see also item X26), as this information may have an effect on user self-selection, user expectation and may also bias results.

1 2 3 4 5

subitem not at all important ○ ○ ○ ○ ○ essential

Does your paper address subitem 4a-iii?

Copy and paste relevant sections from the manuscript (include quotes in quotation marks "like this" to indicate direct quotes from your manuscript), or elaborate on this item by providing additional information not in the ms, or briefly explain why the item is not applicable/relevant for your study

We state: "Those interested could register for the intervention via the intervention website, which provided detailed information about the program and the research study." and "Informed consent was obtained online at the time of enrolment prior to the pre-intervention assessment."

4b) Settings and locations where the data were collected

Does your paper address CONSORT subitem 4b? *

Copy and paste relevant sections from the manuscript (include quotes in quotation marks "like this" to indicate direct quotes from your manuscript), or elaborate on this item by providing additional information not in the ms, or briefly explain why the item is not applicable/relevant for your study

We state: "The assessments took place on three occasions: (1) pre-intervention (September 2022), (2) post-intervention (October 2022), and (3) at the 6-month follow-up (May 2023). Informed consent was obtained online at the time of enrolment prior to the pre-intervention assessment. Participants' self-reported data and data on the use of the program were collected using a secure platform, Iterapi, which hosted the program [16]."

4b-i) Report if outcomes were (self-)assessed through online questionnaires

Clearly report if outcomes were (self-)assessed through online questionnaires (as common in web-based trials) or otherwise.

1 2 3 4 5

subitem not at all important ○ ○ ○ ○ ○ essential

Does your paper address subitem 4b-i? *

Copy and paste relevant sections from the manuscript (include quotes in quotation marks "like this" to indicate direct quotes from your manuscript), or elaborate on this item by providing additional information not in the ms, or briefly explain why the item is not applicable/relevant for your study

We state: "Participants' self-reported data and data on the use of the program were collected using a secure platform, Iterapi, which hosted the program [16]."

4b-ii) Report how institutional affiliations are displayed

Report how institutional affiliations are displayed to potential participants [on ehealth media], as affiliations with prestigious hospitals or universities may affect volunteer rates, use, and reactions with regards to an intervention.(Not a required item – describe only if this may bias results)

1 2 3 4 5

subitem not at all important ○ ○ ○ ○ ○ essential

Does your paper address subitem 4b-ii?

Copy and paste relevant sections from the manuscript (include quotes in quotation marks "like this" to indicate direct quotes from your manuscript), or elaborate on this item by providing additional information not in the ms, or briefly explain why the item is not applicable/relevant for your study

Jūsų atsakymas

5) The interventions for each group with sufficient details to allow replication, including how and when they were actually administered

5-i) Mention names, credential, affiliations of the developers, sponsors, and owners

Mention names, credential, affiliations of the developers, sponsors, and owners [6] (if authors/evaluators are owners or developer of the software, this needs to be declared in a “Conflict of interest” section or mentioned elsewhere in the manuscript).

1 2 3 4 5

subitem not at all important ○ ○ ○ ○ ○ essential

Does your paper address subitem 5-i?

Copy and paste relevant sections from the manuscript (include quotes in quotation marks "like this" to indicate direct quotes from your manuscript), or elaborate on this item by providing additional information not in the ms, or briefly explain why the item is not applicable/relevant for your study

Jūsų atsakymas

5-ii) Describe the history/development process

Describe the history/development process of the application and previous formative evaluations (e.g., focus groups, usability testing), as these will have an impact on adoption/use rates and help with interpreting results.

1 2 3 4 5

subitem not at all important ○ ○ ○ ○ ○ essential

Does your paper address subitem 5-ii?

Copy and paste relevant sections from the manuscript (include quotes in quotation marks "like this" to indicate direct quotes from your manuscript), or elaborate on this item by providing additional information not in the ms, or briefly explain why the item is not applicable/relevant for your study

We state: "FOREST+ is a 6-week stress recovery program specifically designed in close collaboration with healthcare professionals for healthcare workers in Lithuania [5]. FOREST+ was developed as a modification of FOREST for nurses [4,14]."

5-iii) Revisions and updating

Revisions and updating. Clearly mention the date and/or version number of the application/intervention (and comparator, if applicable) evaluated, or describe whether the intervention underwent major changes during the evaluation process, or whether the development and/or content was “frozen” during the trial. Describe dynamic components such as news feeds or changing content which may have an impact on the replicability of the intervention (for unexpected events see item 3b).

1 2 3 4 5

subitem not at all important ○ ○ ○ ○ ○ essential

Does your paper address subitem 5-iii?

Copy and paste relevant sections from the manuscript (include quotes in quotation marks "like this" to indicate direct quotes from your manuscript), or elaborate on this item by providing additional information not in the ms, or briefly explain why the item is not applicable/relevant for your study

Jūsų atsakymas

5-iv) Quality assurance methods

Provide information on quality assurance methods to ensure accuracy and quality of information provided [1], if applicable.

1 2 3 4 5

subitem not at all important ○ ○ ○ ○ ○ essential

Does your paper address subitem 5-iv?

Copy and paste relevant sections from the manuscript (include quotes in quotation marks "like this" to indicate direct quotes from your manuscript), or elaborate on this item by providing additional information not in the ms, or briefly explain why the item is not applicable/relevant for your study

Jūsų atsakymas

5-v) Ensure replicability by publishing the source code, and/or providing screenshots/screen-capture video, and/or providing flowcharts of the algorithms used

Ensure replicability by publishing the source code, and/or providing screenshots/screen-capture video, and/or providing flowcharts of the algorithms used. Replicability (i.e., other researchers should in principle be able to replicate the study) is a hallmark of scientific reporting.

1 2 3 4 5

subitem not at all important ○ ○ ○ ○ ○ essential

Does your paper address subitem 5-v?

Copy and paste relevant sections from the manuscript (include quotes in quotation marks "like this" to indicate direct quotes from your manuscript), or elaborate on this item by providing additional information not in the ms, or briefly explain why the item is not applicable/relevant for your study

Jūsų atsakymas

5-vi) Digital preservation

Digital preservation: Provide the URL of the application, but as the intervention is likely to change or disappear over the course of the years; also make sure the intervention is archived (Internet Archive, webcitation.org, and/or publishing the source code or screenshots/videos alongside the article). As pages behind login screens cannot be archived, consider creating demo pages which are accessible without login.

1 2 3 4 5

subitem not at all important ○ ○ ○ ○ ○ essential

Does your paper address subitem 5-vi?

Copy and paste relevant sections from the manuscript (include quotes in quotation marks "like this" to indicate direct quotes from your manuscript), or elaborate on this item by providing additional information not in the ms, or briefly explain why the item is not applicable/relevant for your study

Jūsų atsakymas

5-vii) Access

Access: Describe how participants accessed the application, in what setting/context, if they had to pay (or were paid) or not, whether they had to be a member of specific group. If known, describe how participants obtained “access to the platform and Internet” [1]. To ensure access for editors/reviewers/readers, consider to provide a “backdoor” login account or demo mode for reviewers/readers to explore the application (also important for archiving purposes, see vi).

1 2 3 4 5

subitem not at all important ○ ○ ○ ○ ○ essential

Does your paper address subitem 5-vii? *

Copy and paste relevant sections from the manuscript (include quotes in quotation marks "like this" to indicate direct quotes from your manuscript), or elaborate on this item by providing additional information not in the ms, or briefly explain why the item is not applicable/relevant for your study

We state: "After completing the registration form and the pre-intervention questionnaire, participants were contacted for a brief phone interview to ensure that they met the eligibility criteria for the study: (1) currently working in a health care facility; (2) adult (>18 years); (3) comprehend Lithuanian; (4) access to a device with internet connection. Certain exclusion criteria have also been identified: (1) high risk of suicide; (2) acute psychiatric crisis; (3) exposure to the current interpersonal violence. Participants who did not meet the eligibility criteria (n=9) were referred elsewhere for psychological support if needed." and "Eligible participants were randomized (1:1) by an independent researcher into two study groups: a standard intervention group or a tailored prompts group, using built-in randomization functionality in the hosting platform. Before registering for the study, participants were informed that the intervention would be provided either with tailored prompts or without. Both groups started using the program after randomization in October 2022."

5-viii) Mode of delivery, features/functionalities/components of the intervention and comparator, and the theoretical framework

Describe mode of delivery, features/functionalities/components of the intervention and comparator, and the theoretical framework [6] used to design them (instructional strategy [1], behaviour change techniques, persuasive features, etc., see e.g., [7, 8] for terminology). This includes an in-depth description of the content (including where it is coming from and who developed it) [1],” whether [and how] it is tailored to individual circumstances and allows users to track their progress and receive feedback” [6]. This also includes a description of communication delivery channels and – if computer-mediated communication is a component – whether communication was synchronous or asynchronous [6]. It also includes information on presentation strategies [1], including page design principles, average amount of text on pages, presence of hyperlinks to other resources, etc. [1].

1 2 3 4 5

subitem not at all important ○ ○ ○ ○ ○ essential

Does your paper address subitem 5-viii? *

Copy and paste relevant sections from the manuscript (include quotes in quotation marks "like this" to indicate direct quotes from your manuscript), or elaborate on this item by providing additional information not in the ms, or briefly explain why the item is not applicable/relevant for your study

We state: "FOREST+ is a 6-week stress recovery program specifically designed in close collaboration with healthcare professionals for healthcare workers in Lithuania [5]. FOREST+ was developed as a modification of FOREST for nurses [4,14]. The FOREST+ program consists of six modules, opened on a weekly schedule: (1) Introduction; (2) Psychological detachment; (3) Distancing; (4) Mastery; (5) Control; and (6) Keeping the change alive. The modules provide psychoeducational information based on the principles of CBT, mindfulness, and four components of stress recovery (mastery, control, psychological detachment, and distancing), as well as various exercises such as mindfulness recordings and self-assessment of bodily tension and stress. The user interface of the program is shown in Figure 1. The language of the program was Lithuanian, and the content of the modules is described in Table 2."

5-ix) Describe use parameters

Describe use parameters (e.g., intended “doses” and optimal timing for use). Clarify what instructions or recommendations were given to the user, e.g., regarding timing, frequency, heaviness of use, if any, or was the intervention used ad libitum.

1 2 3 4 5

subitem not at all important ○ ○ ○ ○ ○ essential

Does your paper address subitem 5-ix?

Copy and paste relevant sections from the manuscript (include quotes in quotation marks "like this" to indicate direct quotes from your manuscript), or elaborate on this item by providing additional information not in the ms, or briefly explain why the item is not applicable/relevant for your study

Jūsų atsakymas

5-x) Clarify the level of human involvement

Clarify the level of human involvement (care providers or health professionals, also technical assistance) in the e-intervention or as co-intervention (detail number and expertise of professionals involved, if any, as well as “type of assistance offered, the timing and frequency of the support, how it is initiated, and the medium by which the assistance is delivered”. It may be necessary to distinguish between the level of human involvement required for the trial, and the level of human involvement required for a routine application outside of a RCT setting (discuss under item 21 – generalizability).

1 2 3 4 5

subitem not at all important ○ ○ ○ ○ ○ essential

Does your paper address subitem 5-x?

Copy and paste relevant sections from the manuscript (include quotes in quotation marks "like this" to indicate direct quotes from your manuscript), or elaborate on this item by providing additional information not in the ms, or briefly explain why the item is not applicable/relevant for your study

We state: "The program is delivered with therapist support using a low-intensity approach. Once the users had completed a weekly worksheet, they received personalized feedback from their therapist. Participants could also contact their therapist via text messaging

integrated into the intervention platform. In addition to communication with the therapist, clients received support from administrators via emails to keep them informed about the progress of the program and to remind them to join a new module or complete worksheets. Administrators also called the participants for a short interview before the intervention and in the middle of the program to inform them of the progress and answer any questions they may have on the use of the intervention."

5-xi) Report any prompts/reminders used

Report any prompts/reminders used: Clarify if there were prompts (letters, emails, phone calls, SMS) to use the application, what triggered them, frequency etc. It may be necessary to distinguish between the level of prompts/reminders required for the trial, and the level of prompts/reminders for a routine application outside of a RCT setting (discuss under item 21 – generalizability).

	1	2	3	4	5	
subitem not at all important	○	○	○	○	○	essential

Does your paper address subitem 5-xi? *

Copy and paste relevant sections from the manuscript (include quotes in quotation marks "like this" to indicate direct quotes from your manuscript), or elaborate on this item by providing additional information not in the ms, or briefly explain why the item is not applicable/relevant for your study

We state: "In addition to communication with the therapist, clients received support from administrators via emails to keep them informed about the progress of the program and to remind them to join a new module or complete worksheets. Administrators also called the participants for a short interview before the intervention and in the middle of the program to inform them of the progress and answer any questions they may have on the use of the intervention." and "In the tailored prompts group, client-administrator communication was supplemented by reminders via the short messages service (SMS). Prompts consisted of a short text asking if the participant had already taken the time to unwind and a link to the mindfulness exercises for that week. SMS prompts were tailored to each participant in the group during a phone interview with the study administrator before using the program. Participants were asked how actively they planned to engage with the program and whether they would need short text message reminders to achieve this. When participants indicated that supplementary prompts would be necessary, they were asked about preferences of prompt frequency (i.e., once a week, twice a week, every workday, or every workday twice a day) and timing (before noon – sometime in the morning or afternoon – sometime in the evening). Participants in the standard intervention group did not schedule a plan for the tailored prompts and did not receive them."

5-xii) Describe any co-interventions (incl. training/support)

Describe any co-interventions (incl. training/support): Clearly state any interventions that are provided in addition to the targeted eHealth intervention, as ehealth intervention may not be designed as stand-alone intervention. This includes training sessions and support [1]. It may be necessary to distinguish between the level of training required for the trial, and the level of training for a routine application outside of a RCT setting (discuss under item 21 – generalizability.

	1	2	3	4	5	
subitem not at all important	○	○	○	○	○	essential

Does your paper address subitem 5-xii? *

Copy and paste relevant sections from the manuscript (include quotes in quotation marks "like this" to indicate direct quotes from your manuscript), or elaborate on this item by providing additional information not in the ms, or briefly explain why the item is not applicable/relevant for your study

No co-interventions were provided in the context of the current study, except for those used by participants themselves as not part of the current study. Participation in other interventions is reported in the Methods section.

6a) Completely defined pre-specified primary and secondary outcome measures, including how and when they were assessed

Does your paper address CONSORT subitem 6a? *

Copy and paste relevant sections from the manuscript (include quotes in quotation marks "like this" to indicate direct quotes from your manuscript), or elaborate on this item by providing additional information not in the ms, or briefly explain why the item is not applicable/relevant for your study

We state: "The assessments took place on three occasions: (1) pre-intervention (September 2022), (2) post-intervention (October 2022), and (3) at the 6-month follow-up (May 2023). Informed consent was obtained online at the time of enrolment prior to the pre-intervention assessment. Participants' self-reported data and data on the use of the program were collected using a secure platform, Iterapi, which hosted the program [16]."
Also, we completely describe primary and secondary measures included in the study:
"Recovery from stress
An overall recovery from stress experience was measured by the Recovery Experiences Questionnaire (REQ)[15]. The REQ consists of 16 items (e.g., "I take time for leisure"). Each of the items can be evaluated on a 5-point Likert-type scale, where 1 is "Totally Disagree" and 5 is "Totally Agree"; scores are calculated by adding up the points, with higher scores indicating a more pronounced stress recovery experience. The REQ showed good validity in the Lithuanian sample [18]. In this study, the REQ McDonald's Omega was excellent at all three measurements: ωT1=0.87, ωT2=0.90, ωT3=0.88.
Perceived stress
The brief Perceived Stress Scale (PSS-4) [19] was used to measure stress levels. The PSS-4 consists of four questions (e.g., "In the last month, how often have you felt that you were unable to control the important things in your life?"). The questions are answered on a 5-point Likert-type scale, where 0 is "Never" and 4 is "Very often"; with the final score calculated by summing all the responses (reversing the scores of items 2 and 3). Higher scores show higher levels of perceived stress. Studies show good psychometric properties of the PSS-4 scale [19]. The PSS-4 McDonald's Omega in the current sample was moderate but close to the acceptable level of 0.70 [20]: ωT1=0.67, ωT2=0.69, ωT3=0.61.
Anxiety and depression
Participants' levels of anxiety and depression were assessed using the four-item Patient Health Questionnaire (PHQ-4)[21]. The PHQ-4 consists of two items to assess depression (e.g., "Feeling down, depressed or hopeless") and two to assess anxiety (e.g., "Feeling nervous, anxious or on edge"). The respondent rates how much each item bothered them on a 4-point Likert-type scale, where 0 is "Not at all" and 3 is "Nearly every day". Adding the item scores for each subscale gives an estimated level of depression or anxiety. Research has shown good PHQ-4 psychometric properties in the Lithuanian sample [18]. The PHQ-4 Cronbach's alpha in the current sample for the anxiety subscale was acceptable: αT1=0.70, αT2=0.74, αT3=0.78; as well as for the depression subscale: αT1=0.71, αT2=0.82, αT3=0.80.
Psychological well-being
The psychological well-being was measured with the World Health Organization-Five Well-Being Index (WHO-5). WHO-5 consists of five items (e.g., "I have felt calm and relaxed"), which are rated on a 6-point Likert-type scale, where 0 is "At no time" and 5 is "All the time". The final percentage well-being score (ranging from 0 to 100) is calculated by summing the item scores and then multiplying the raw score by 4. Higher final scores indicate better psychological well-being. The WHO-5 has been translated and used in Lithuanian sample studies [22]. The WHO-5 McDonald's Omega in the current sample was good: ωT1=0.83, ωT2=0.86, ωT3=0.82."

6a-i) Online questionnaires: describe if they were validated for online use and apply CHERRIES items to describe how the questionnaires were designed/deployed

If outcomes were obtained through online questionnaires, describe if they were validated for online use and apply CHERRIES items to describe how the questionnaires were designed/deployed [9].

	1	2	3	4	5	
subitem not at all important	○	○	○	○	○	essential

Does your paper address subitem 6a-i?

Copy and paste relevant sections from manuscript text

Jūsų atsakymas

6a-ii) Describe whether and how "use" (including intensity of use/dosage) was defined/measured/monitored

Describe whether and how "use" (including intensity of use/dosage) was defined/measured/monitored (logins, logfile analysis, etc.). Use/adoption metrics are important process outcomes that should be reported in any ehealth trial.

	1	2	3	4	5	
subitem not at all important	○	○	○	○	○	essential

Does your paper address subitem 6a-ii?

Copy and paste relevant sections from manuscript text

We state: "During the pre-intervention assessment, participants were asked how actively they expected to use the program, where 1 was "I will not use it" and 10 was "I will use it a lot". At the post-intervention evaluation, participants were asked how actively they actually had used the program (1="I did not use it" and 10="I used it a lot") and how much time on average per week they had managed to devote to the program (1="Not at all" and 6=">2 hours"). The congruence of use expectations was measured by subtracting the score reflecting the pre-intervention expected usage from the post-intervention assessment of subjective usage. Moreover, participants' usage information was exported directly from the platform. Data were collected on the number of logins, modules opened and completed (from 0 to 6), exercises completed (from 0 to 19), messages received from the therapist, and messages sent to the therapist."

6a-iii) Describe whether, how, and when qualitative feedback from participants was obtained

Describe whether, how, and when qualitative feedback from participants was obtained (e.g., through emails, feedback forms, interviews, focus groups).

	1	2	3	4	5	
subitem not at all important	○	○	○	○	○	essential

Does your paper address subitem 6a-iii?

Copy and paste relevant sections from manuscript text

Jūsų atsakymas

6b) Any changes to trial outcomes after the trial commenced, with reasons

Does your paper address CONSORT subitem 6b? *

Copy and paste relevant sections from the manuscript (include quotes in quotation marks "like this" to indicate direct quotes from your manuscript), or elaborate on this item by providing additional information not in the ms, or briefly explain why the item is not applicable/relevant for your study

No changes to trial outcomes after the trial commenced were made.

7a) How sample size was determined

NPT: When applicable, details of whether and how the clustering by care provides or centers was addressed

7a-i) Describe whether and how expected attrition was taken into account when calculating the sample size

Describe whether and how expected attrition was taken into account when calculating the sample size.

	1	2	3	4	5	
subitem not at all important	○	○	○	○	○	essential

Does your paper address subitem 7a-i?

Copy and paste relevant sections from manuscript title (include quotes in quotation marks "like this" to indicate direct quotes from your manuscript), or elaborate on this item by providing additional information not in the ms, or briefly explain why the item is not applicable/relevant for your study

Jūsų atsakymas

7b) When applicable, explanation of any interim analyses and stopping guidelines

Does your paper address CONSORT subitem 7b? *

Copy and paste relevant sections from the manuscript (include quotes in quotation marks "like this" to indicate direct quotes from your manuscript), or elaborate on this item by providing additional information not in the ms, or briefly explain why the item is not applicable/relevant for your study

This item is not applicable.

8a) Method used to generate the random allocation sequence

NPT: When applicable, how care providers were allocated to each trial group

Does your paper address CONSORT subitem 8a? *

Copy and paste relevant sections from the manuscript (include quotes in quotation marks "like this" to indicate direct quotes from your manuscript), or elaborate on this item by providing additional information not in the ms, or briefly explain why the item is not applicable/relevant for your study

We state: "Eligible participants were randomized (1:1) by an independent researcher into two study groups: a standard intervention group or a tailored prompts group, using built-in randomization functionality in the hosting platform."

8b) Type of randomisation; details of any restriction (such as blocking and block size)

Does your paper address CONSORT subitem 8b? *

Copy and paste relevant sections from the manuscript (include quotes in quotation marks "like this" to indicate direct quotes from your manuscript), or elaborate on this item by providing additional information not in the ms, or briefly explain why the item is not applicable/relevant for your study

We state: "Eligible participants were randomized (1:1) by an independent researcher into two study groups: a standard intervention group or a tailored prompts group, using built-in randomization functionality in the hosting platform."

9) Mechanism used to implement the random allocation sequence (such as sequentially numbered containers), describing any steps taken to conceal the sequence until interventions were assigned

Does your paper address CONSORT subitem 9? *

Copy and paste relevant sections from the manuscript (include quotes in quotation marks "like this" to indicate direct quotes from your manuscript), or elaborate on this item by providing additional information not in the ms, or briefly explain why the item is not applicable/relevant for your study

We state: "Eligible participants were randomized (1:1) by an independent researcher into two study groups: a standard intervention group or a tailored prompts group, using built-in randomization functionality in the hosting platform."

10) Who generated the random allocation sequence, who enrolled participants, and who assigned participants to interventions

Does your paper address CONSORT subitem 10? *

Copy and paste relevant sections from the manuscript (include quotes in quotation marks "like this" to indicate direct quotes from your manuscript), or elaborate on this item by providing additional information not in the ms, or briefly explain why the item is not applicable/relevant for your study

We state: "Eligible participants were randomized (1:1) by an independent researcher into two study groups: a standard intervention group or a tailored prompts group, using built-in randomization functionality in the hosting platform."

11a) If done, who was blinded after assignment to interventions (for example, participants, care providers, those assessing outcomes) and how

NPT: Whether or not administering co-interventions were blinded to group assignment

11a-i) Specify who was blinded, and who wasn't

Specify who was blinded, and who wasn't. Usually, in web-based trials it is not possible to blind the participants [1, 3] (this should be clearly acknowledged), but it may be possible to blind outcome assessors, those doing data analysis or those administering co-interventions (if any).

	1	2	3	4	5	
subitem not at all important	○	○	○	○	○	essential

Does your paper address subitem 11a-i? *

Copy and paste relevant sections from the manuscript (include quotes in quotation marks "like this" to indicate direct quotes from your manuscript), or elaborate on this item by providing additional information not in the ms, or briefly explain why the item is not applicable/relevant for your study

We state: "Before registering for the study, participants were informed that the intervention would be provided either with tailored prompts or without." No blinding was done after the assignment to interventions.

11a-ii) Discuss e.g., whether participants knew which intervention was the "intervention of interest" and which one was the "comparator"

Informed consent procedures (4a-ii) can create biases and certain expectations - discuss e.g., whether participants knew which intervention was the "intervention of interest" and which one was the "comparator".

	1	2	3	4	5	
subitem not at all important	○	○	○	○	○	essential

Does your paper address subitem 11a-ii?

Copy and paste relevant sections from the manuscript (include quotes in quotation marks "like this" to indicate direct quotes from your manuscript), or elaborate on this item by providing additional information not in the ms, or briefly explain why the item is not applicable/relevant for your study

Jūsų atsakymas

11b) If relevant, description of the similarity of interventions

(this item is usually not relevant for ehealth trials as it refers to similarity of a placebo or sham intervention to a active medication/intervention)

Does your paper address CONSORT subitem 11b? *

Copy and paste relevant sections from the manuscript (include quotes in quotation marks "like this" to indicate direct quotes from your manuscript), or elaborate on this item by providing additional information not in the ms, or briefly explain why the item is not applicable/relevant for your study

The item is not applicable.

12a) Statistical methods used to compare groups for primary and secondary outcomes

NPT: When applicable, details of whether and how the clustering by care providers or centers was addressed

Does your paper address CONSORT subitem 12a? *

Copy and paste relevant sections from the manuscript (include quotes in quotation marks "like this" to indicate direct quotes from your manuscript), or elaborate on this item by providing additional information not in the ms, or briefly explain why the item is not applicable/relevant for your study

We state: "To assess the effect of the program on primary (stress recovery) and secondary outcomes (perceived stress levels, anxiety, depression, psychological well-being), a Latent Change Modeling (LCM) approach [23] using Mplus 8.8 [24] was carried out. To estimate the within-group effects of the standard intervention and the intervention supplemented by tailored prompts, a series of multi-group LCMs were performed, reporting changes in primary and secondary outcomes from pre-intervention to post-intervention and from pre-intervention to 6-month follow-up in each group separately. To calculate the between-group effects, a series of conditional LCM was computed in a full sample by regressing the intervention condition (0=standard intervention group; 1=tailored prompts group) on the changes in outcome scores. Moreover, we ran a series of univariate regression analyses to explore whether prompt timing and frequency were associated with changes in outcomes in the tailored prompts group."

12a-i) Imputation techniques to deal with attrition / missing values

Imputation techniques to deal with attrition / missing values: Not all participants will use the intervention/comparator as intended and attrition is typically high in ehealth trials. Specify how participants who did not use the application or dropped out from the trial were treated in the statistical analysis (a complete case analysis is strongly discouraged, and simple imputation techniques such as LOCF may also be problematic [4]).

	1	2	3	4	5	
subitem not at all important	○	○	○	○	○	essential

Does your paper address subitem 12a-i? *

Copy and paste relevant sections from the manuscript (include quotes in quotation marks "like this" to indicate direct quotes from your manuscript), or elaborate on this item by providing additional information not in the ms, or briefly explain why the item is not applicable/relevant for your study

We state: "The Full Information Maximum Likelihood (FIML) algorithm was used for handling the missing data."

12b) Methods for additional analyses, such as subgroup analyses and adjusted analyses

Does your paper address CONSORT subitem 12b? *

Copy and paste relevant sections from the manuscript (include quotes in quotation marks "like this" to indicate direct quotes from your manuscript), or elaborate on this item by providing additional information not in the ms, or briefly explain why the item is not applicable/relevant for your study

No additional analyses, such as subgroup analyses and adjusted analyses, were performed.

X26) REB/IRB Approval and Ethical Considerations [recommended as subheading under "Methods"] (not a CONSORT item)

X26-i) Comment on ethics committee approval

	1	2	3	4	5	
subitem not at all important	○	○	○	○	○	essential

Does your paper address subitem X26-i?

Copy and paste relevant sections from the manuscript (include quotes in quotation marks "like this" to indicate direct quotes from your manuscript), or elaborate on this item by providing additional information not in the ms, or briefly explain why the item is not applicable/relevant for your study

Jūsų atsakymas

x26-ii) Outline informed consent procedures

Outline informed consent procedures e.g., if consent was obtained offline or online (how? Checkbox, etc.?), and what information was provided (see 4a-ii). See [6] for some items to be included in informed consent documents.

	1	2	3	4	5	
subitem not at all important	○	○	○	○	○	essential

Does your paper address subitem X26-ii?

Copy and paste relevant sections from the manuscript (include quotes in quotation marks "like this" to indicate direct quotes from your manuscript), or elaborate on this item by providing additional information not in the ms, or briefly explain why the item is not applicable/relevant for your study

We state: "Informed consent was obtained online at the time of enrolment prior to the pre-intervention assessment." and "Those interested could register for the intervention via the intervention website, which provided detailed information about the program and the research study."

X26-iii) Safety and security procedures

Safety and security procedures, incl. privacy considerations, and any steps taken to reduce the likelihood or detection of harm (e.g., education and training, availability of a hotline)

	1	2	3	4	5	
subitem not at all important	○	○	○	○	○	essential

Does your paper address subitem X26-iii?

Copy and paste relevant sections from the manuscript (include quotes in quotation marks "like this" to indicate direct quotes from your manuscript), or elaborate on this item by providing additional information not in the ms, or briefly explain why the item is not applicable/relevant for your study

Jūsų atsakymas

RESULTS

13a) For each group, the numbers of participants who were randomly assigned, received intended treatment, and were analysed for the primary outcome

NPT: The number of care providers or centers performing the intervention in each group and the number of patients treated by each care provider in each center

Does your paper address CONSORT subitem 13a? *

Copy and paste relevant sections from the manuscript (include quotes in quotation marks "like this" to indicate direct quotes from your manuscript), or elaborate on this item by providing additional information not in the ms, or briefly explain why the item is not applicable/relevant for your study

We state: "The study flowchart is shown in Figure 2. More than one hundred individuals registered to participate in the intervention. In total, 91 individuals met eligibility criteria and were randomized to one of the two study groups: tailored prompts group (TG; n=46) or standard intervention group (SG; n=45). Four of the participants did not log in to the program (two from each group) and were therefore not included in the data analysis. The final data analysis included 87 participants (nTG=44, nSG=43)."

13b) For each group, losses and exclusions after randomisation, together with reasons

Does your paper address CONSORT subitem 13b? (NOTE: Preferably, this is shown in a CONSORT flow diagram) *

Copy and paste relevant sections from the manuscript (include quotes in quotation marks "like this" to indicate direct quotes from your manuscript), or elaborate on this item by providing additional information not in the ms, or briefly explain why the item is not applicable/relevant for your study

We state: "The study flowchart is shown in Figure 2. More than one hundred individuals registered to participate in the intervention. In total, 91 individuals met eligibility criteria and were randomized to one of the two study groups: tailored prompts group (TG; n=46) or standard intervention group (SG; n=45). Four of the participants did not log in to the program (two from each group) and were therefore not included in the data analysis. The final data analysis included 87 participants (nTG=44, nSG=43)."

13b-i) Attrition diagram

Strongly recommended: An attrition diagram (e.g., proportion of participants still logging in or using the intervention/comparator in each group plotted over time, similar to a survival curve) or other figures or tables demonstrating usage/dose/engagement.

	1	2	3	4	5	
subitem not at all important	○	○	○	○	○	essential

Does your paper address subitem 13b-i?

Copy and paste relevant sections from the manuscript or cite the figure number if applicable (include quotes in quotation marks "like this" to indicate direct quotes from your manuscript), or elaborate on this item by providing additional information not in the ms, or briefly explain why the item is not applicable/relevant for your study

We state: "Around one-fifth of participants (n=18, 21%) from the full sample fully completed all six program modules (see Table 3); as previously, no difference between the tailored prompts group (M=3.25, SD=2.15) and standard intervention group (M=2.91, SD=2.38) in completed modules was observed (t(85)=-0.71, P=.48)."

14a) Dates defining the periods of recruitment and follow-up

Does your paper address CONSORT subitem 14a? *

Copy and paste relevant sections from the manuscript (include quotes in quotation marks "like this" to indicate direct quotes from your manuscript), or elaborate on this item by providing additional information not in the ms, or briefly explain why the item is not applicable/relevant for your study

We state: "Both groups started using the program after randomization in October 2022. The assessments took place on three occasions: (1) pre-intervention (September 2022), (2) post-intervention (October 2022), and (3) at the 6-month follow-up (May 2023)."

14a-i) Indicate if critical "secular events" fell into the study period

Indicate if critical "secular events" fell into the study period, e.g., significant changes in Internet resources available or "changes in computer hardware or Internet delivery resources"

	1	2	3	4	5	
subitem not at all important	○	○	○	○	○	essential

Does your paper address subitem 14a-i?

Copy and paste relevant sections from the manuscript (include quotes in quotation marks "like this" to indicate direct quotes from your manuscript), or elaborate on this item by providing additional information not in the ms, or briefly explain why the item is not applicable/relevant for your study

Jūsų atsakymas

14b) Why the trial ended or was stopped (early)

Does your paper address CONSORT subitem 14b? *

Copy and paste relevant sections from the manuscript (include quotes in quotation marks "like this" to indicate direct quotes from your manuscript), or elaborate on this item by providing additional information not in the ms, or briefly explain why the item is not applicable/relevant for your study

This item is not applicable.

15) A table showing baseline demographic and clinical characteristics for each group

NPT: When applicable, a description of care providers (case volume, qualification, expertise, etc.) and centers (volume) in each group

Does your paper address CONSORT subitem 15? *

Copy and paste relevant sections from the manuscript (include quotes in quotation marks "like this" to indicate direct quotes from your manuscript), or elaborate on this item by providing additional information not in the ms, or briefly explain why the item is not applicable/relevant for your study

We state: "The participants included in the study were almost exclusively female (n=86, 99%). Participants ranged in age from 19 to 68 years (M=39.61, SD=11.49). Around a quarter of the participants were licensed medical doctors (n=24, 28%), 9% (n=8) were resident doctors, 31% (n=27) – nurses, and 32% (n=28) – other healthcare workers (psychologists, social workers, complementary and alternative health professionals, public health professionals, oral care professionals). The standard intervention group and the tailored prompts groups did not differ significantly in the pre-intervention (baseline) measures of sociodemographic, work-related, psychological support, and mental health factors. The sample characteristics of the groups at baseline are shown in Table 1."

15-i) Report demographics associated with digital divide issues

In ehealth trials it is particularly important to report demographics associated with digital divide issues, such as age, education, gender, social-economic status, computer/Internet/ehealth literacy of the participants, if known.

	1	2	3	4	5	
subitem not at all important	○	○	○	○	○	essential

Does your paper address subitem 15-i? *

Copy and paste relevant sections from the manuscript (include quotes in quotation marks "like this" to indicate direct quotes from your manuscript), or elaborate on this item by providing additional information not in the ms, or briefly explain why the item is not applicable/relevant for your study

Information regarding digital divice issues or skills was not obtained.

16) For each group, number of participants (denominator) included in each analysis and whether the analysis was by original assigned groups

16-i) Report multiple "denominators" and provide definitions

Report multiple "denominators" and provide definitions: Report N's (and effect sizes) "across a range of study participation [and use] thresholds" [1], e.g., N exposed, N consented, N used more than x times, N used more than y weeks, N participants "used" the intervention/comparator at specific pre-defined time points of interest (in absolute and relative numbers per group). Always clearly define "use" of the intervention.

	1	2	3	4	5	
subitem not at all important	○	○	○	○	○	essential

Does your paper address subitem 16-i? *

Copy and paste relevant sections from the manuscript (include quotes in quotation marks "like this" to indicate direct quotes from your manuscript), or elaborate on this item by providing additional information not in the ms, or briefly explain why the item is not applicable/relevant for your study

We state: The distribution of message preferences for all participants who took part in the pre-intervention interviews (n=47) is shown in Appendix Table 1.", "Table 3. Number of program modules completed (N=87)." and "The trajectories of outcomes in each group are shown in Figure 2, and Table 4 presents outcome means and standard deviations. Within-group effect sizes are presented in Table 5."

16-ii) Primary analysis should be intent-to-treat

Primary analysis should be intent-to-treat, secondary analyses could include comparing only "users", with the appropriate caveats that this is no longer a randomized sample (see 18-i).

	1	2	3	4	5	
subitem not at all important	○	○	○	○	○	essential

Does your paper address subitem 16-ii?

Copy and paste relevant sections from the manuscript (include quotes in quotation marks "like this" to indicate direct quotes from your manuscript), or elaborate on this item by providing additional information not in the ms, or briefly explain why the item is not applicable/relevant for your study

Jūsų atsakymas

17a) For each primary and secondary outcome, results for each group, and the estimated effect size and its precision (such as 95% confidence interval)

Does your paper address CONSORT subitem 17a? *

Copy and paste relevant sections from the manuscript (include quotes in quotation marks "like this" to indicate direct quotes from your manuscript), or elaborate on this item by providing additional information not in the ms, or briefly explain why the item is not applicable/relevant for your study

We state: " "The trajectories of outcomes in each group are shown in Figure 2, and Table 4 presents outcome means and standard deviations. Within-group effect sizes and 95% C.I. are presented in Table 5."

17a-i) Presentation of process outcomes such as metrics of use and intensity of use

In addition to primary/secondary (clinical) outcomes, the presentation of process outcomes such as metrics of use and intensity of use (dose, exposure) and their operational definitions is critical. This does not only refer to metrics of attrition (13-b) (often a binary variable), but also to more continuous exposure metrics such as "average session length". These must be accompanied by a technical description how a metric like a "session" is defined (e.g., timeout after idle time) [1] (report under item 6a).

	1	2	3	4	5	
subitem not at all important	○	○	○	○	○	essential

Does your paper address subitem 17a-i?

Copy and paste relevant sections from the manuscript (include quotes in quotation marks "like this" to indicate direct quotes from your manuscript), or elaborate on this item by providing additional information not in the ms, or briefly explain why the item is not applicable/relevant for your study

Metrics of use and intensity of use are presented in detail in section Use and Support Received.

17b) For binary outcomes, presentation of both absolute and relative effect sizes is recommended

Does your paper address CONSORT subitem 17b? *

Copy and paste relevant sections from the manuscript (include quotes in quotation marks "like this" to indicate direct quotes from your manuscript), or elaborate on this item by providing additional information not in the ms, or briefly explain why the item is not applicable/relevant for your study

No binary outcomes were used in the current study.

18) Results of any other analyses performed, including subgroup analyses and adjusted analyses, distinguishing pre-specified from exploratory

Does your paper address CONSORT subitem 18? *

Copy and paste relevant sections from the manuscript (include quotes in quotation marks "like this" to indicate direct quotes from your manuscript), or elaborate on this item by providing additional information not in the ms, or briefly explain why the item is not applicable/relevant for your study

Detailed description of statistical analysis procedure is provided in the Method section.Exploratory analysis was not performed. No adjustments for baseline variables were made. Subgroup analysis was not performed.

18-i) Subgroup analysis of comparing only users

A subgroup analysis of comparing only users is not uncommon in ehealth trials, but if done, it must be stressed that this is a self-selected sample and no longer an unbiased sample from a randomized trial (see 16-iii).

	1	2	3	4	5	
subitem not at all important	○	○	○	○	○	essential

Does your paper address subitem 18-i?

Copy and paste relevant sections from the manuscript (include quotes in quotation marks "like this" to indicate direct quotes from your manuscript), or elaborate on this item by providing additional information not in the ms, or briefly explain why the item is not applicable/relevant for your study

Jūsų atsakymas

19) All important harms or unintended effects in each group

(for specific guidance see CONSORT for harms)

Does your paper address CONSORT subitem 19? *

Copy and paste relevant sections from the manuscript (include quotes in quotation marks "like this" to indicate direct quotes from your manuscript), or elaborate on this item by providing additional information not in the ms, or briefly explain why the item is not applicable/relevant for your study

Participants did not report any harmful experiences during the study period.

19-i) Include privacy breaches, technical problems

Include privacy breaches, technical problems. This does not only include physical "harm" to participants, but also incidents such as perceived or real privacy breaches [1], technical problems, and other unexpected/unintended incidents. "Unintended effects" also includes unintended positive effects [2].

	1	2	3	4	5	
subitem not at all important	○	○	○	○	○	essential

Does your paper address subitem 19-i?

Copy and paste relevant sections from the manuscript (include quotes in quotation marks "like this" to indicate direct quotes from your manuscript), or elaborate on this item by providing additional information not in the ms, or briefly explain why the item is not applicable/relevant for your study

Jūsų atsakymas

19-ii) Include qualitative feedback from participants or observations from staff/researchers

Include qualitative feedback from participants or observations from staff/researchers, if available, on strengths and shortcomings of the application, especially if they point to unintended/unexpected effects or uses. This includes (if available) reasons for why people did or did not use the application as intended by the developers.

	1	2	3	4	5	
subitem not at all important	○	○	○	○	○	essential

Does your paper address subitem 19-ii?

Copy and paste relevant sections from the manuscript (include quotes in quotation marks "like this" to indicate direct quotes from your manuscript), or elaborate on this item by providing additional information not in the ms, or briefly explain why the item is not applicable/relevant for your study

Jūsų atsakymas

DISCUSSION

22) Interpretation consistent with results, balancing benefits and harms, and considering other relevant evidence

NPT: In addition, take into account the choice of the comparator, lack of or partial blinding, and unequal expertise of care providers or centers in each group

22-i) Restate study questions and summarize the answers suggested by the data, starting with primary outcomes and process outcomes (use)

Restate study questions and summarize the answers suggested by the data, starting with primary outcomes and process outcomes (use).

	1	2	3	4	5	
subitem not at all important	○	○	○	○	○	essential

Does your paper address subitem 22-i? *

Copy and paste relevant sections from the manuscript (include quotes in quotation marks "like this" to indicate direct quotes from your manuscript), or elaborate on this item by providing additional information not in the ms, or briefly explain why the item is not applicable/relevant for your study

Detailed information is provided in the Discussion section. For example: "The aim of this study was to test whether the inclusion of tailored prompts to pursue the individual usage intensity goal would increase engagement and efficacy of ICBT stress recovery intervention for healthcare workers. While users expressed satisfaction with the intervention and received supplementary prompts, results revealed that tailored prompts had no significant effects on usage indicators and were not associated with additional stress recovery, perceived stress, anxiety, depression, or psychological well-being outcomes. The results thus bring new insights to the field of research on internet interventions and call to consider the possible effects of supplementary tailored prompts when designing or testing internet interventions."

22-ii) Highlight unanswered new questions, suggest future research

Highlight unanswered new questions, suggest future research.

	1	2	3	4	5	
subitem not at all important	○	○	○	○	○	essential

Does your paper address subitem 22-ii?

Copy and paste relevant sections from the manuscript (include quotes in quotation marks "like this" to indicate direct quotes from your manuscript), or elaborate on this item by providing additional information not in the ms, or briefly explain why the item is not applicable/relevant for your study

We state: "Thus, further research should test other factors of engagement that might be influenced by including tailored prompts and clarify what works for whom to ensure optimal participant engagement." and "Future research should further explore different factors that influence engagement and the efficacy of internet interventions and the mechanisms driving their success."

20) Trial limitations, addressing sources of potential bias, imprecision, and, if relevant, multiplicity of analyses

20-i) Typical limitations in ehealth trials

Typical limitations in ehealth trials: Participants in ehealth trials are rarely blinded. Ehealth trials often look at a multiplicity of outcomes, increasing risk for a Type I error. Discuss biases due to non-use of the intervention/usability issues, biases through informed consent procedures, unexpected events.

	1	2	3	4	5	
subitem not at all important	○	○	○	○	○	essential

Does your paper address subitem 20-i? *

Copy and paste relevant sections from the manuscript (include quotes in quotation marks "like this" to indicate direct quotes from your manuscript), or elaborate on this item by providing additional information not in the ms, or briefly explain why the item is not applicable/relevant for your study

In the Limitations section, we state: "The results of this study should be viewed in the context of its limitations. Even though previous research has found the tested stress recovery intervention to be effective at post-intervention assessment [4,5], in the current study, no significant changes in the perceived stress at post-intervention were found in comparison to baseline assessment. This could be explained by one of the shortcomings of this study – the modest sample size. As well as by different measures used, as the short version of the PSS scale had relatively low reliability in our study, it may not have captured more nuanced changes. Secondly, the results of this study cannot be generalized to a broader population, as participants were almost exclusively female and healthcare workers. Lastly, the absence of a third control group that did not receive ICBT limits comparison possibilities. Despite the limitations, this novel study provides a further understanding of how internet intervention effects can be influenced by the inclusion of tailored prompts to achieve usage goals."

21) Generalisability (external validity, applicability) of the trial findings

NPT: External validity of the trial findings according to the intervention, comparators, patients, and care providers or centers involved in the trial

21-i) Generalizability to other populations

Generalizability to other populations: In particular, discuss generalizability to a general Internet population, outside of a RCT setting, and general patient population, including applicability of the study results for other organizations

	1	2	3	4	5	
subitem not at all important	○	○	○	○	○	essential

Does your paper address subitem 21-i?

Copy and paste relevant sections from the manuscript (include quotes in quotation marks "like this" to indicate direct quotes from your manuscript), or elaborate on this item by providing additional information not in the ms, or briefly explain why the item is not applicable/relevant for your study

Jūsų atsakymas

21-ii) Discuss if there were elements in the RCT that would be different in a routine application setting

Discuss if there were elements in the RCT that would be different in a routine application setting (e.g., prompts/reminders, more human involvement, training sessions or other co-interventions) and what impact the omission of these elements could have on use, adoption, or outcomes if the intervention is applied outside of a RCT setting.

	1	2	3	4	5	
subitem not at all important	○	○	○	○	○	essential

Does your paper address subitem 21-ii?

Copy and paste relevant sections from the manuscript (include quotes in quotation marks "like this" to indicate direct quotes from your manuscript), or elaborate on this item by providing additional information not in the ms, or briefly explain why the item is not applicable/relevant for your study

Jūsų atsakymas

OTHER INFORMATION

23) Registration number and name of trial registry

Does your paper address CONSORT subitem 23? *

Copy and paste relevant sections from the manuscript (include quotes in quotation marks "like this" to indicate direct quotes from your manuscript), or elaborate on this item by providing additional information not in the ms, or briefly explain why the item is not applicable/relevant for your study

ClinicalTrials.gov, NCT05553210, https://clinicaltrials.gov/study/NCT05553210.

24) Where the full trial protocol can be accessed, if available

Does your paper address CONSORT subitem 24? *

Cite a Multimedia Appendix, other reference, or copy and paste relevant sections from the manuscript (include quotes in quotation marks "like this" to indicate direct quotes from your manuscript), or elaborate on this item by providing additional information not in the ms, or briefly explain why the item is not applicable/relevant for your study

Jovarauskaite L, Dumarkaite A, Truskauskaite-Kuneviciene I, Jovaisiene I, Andersson G, Kazlauskas E. Internet-based stress recovery intervention FOREST for healthcare staff amid COVID-19 pandemic: study protocol for a randomized controlled trial. Trials. 2021;22(1):1-9. doi:10.1186/s13063-021-05512-1

25) Sources of funding and other support (such as supply of drugs), role of funders

Does your paper address CONSORT subitem 25? *

Copy and paste relevant sections from the manuscript (include quotes in quotation marks "like this" to indicate direct quotes from your manuscript), or elaborate on this item by providing additional information not in the ms, or briefly explain why the item is not applicable/relevant for your study

We state: "The project has received funding from the European Regional Development Fund (project No: 01.2.2-LMT-K-718-03-0072) under a grant agreement with the Research Council of Lithuania (LMTLT)."

X27) Conflicts of Interest (not a CONSORT item)

X27-i) State the relation of the study team towards the system being evaluated

In addition to the usual declaration of interests (financial or otherwise), also state the relation of the study team towards the system being evaluated, i.e., state if the authors/evaluators are distinct from or identical with the developers/sponsors of the intervention.

	1	2	3	4	5

subitem not at all important ○ ○ ○ ○ ○ essential

Does your paper address subitem X27-i?

Copy and paste relevant sections from the manuscript (include quotes in quotation marks "like this" to indicate direct quotes from your manuscript), or elaborate on this item by providing additional information not in the ms, or briefly explain why the item is not applicable/relevant for your study

No potential conflict of interest was declared by the author(s).

About the CONSORT EHEALTH checklist

As a result of using this checklist, did you make changes in your manuscript? *

- ○ yes, major changes
- ○ yes, minor changes
- ◉ no

What were the most important changes you made as a result of using this checklist?

No changes, as the manuscript was checked using this checklist before the submission.

How much time did you spend on going through the checklist INCLUDING making changes in your manuscript *

I have spent approximately 1 hour.

As a result of using this checklist, do you think your manuscript has improved? *

- ◉ yes
- ○ no
- ○ Kita:

Would you like to become involved in the CONSORT EHEALTH group?

This would involve for example becoming involved in participating in a workshop and writing an "Explanation and Elaboration" document

- ○ yes
- ○ no
- ○ Kita:

Any other comments or questions on CONSORT EHEALTH

Jūsų atsakymas

STOP - Save this form as PDF before you click submit

To generate a record that you filled in this form, we recommend to generate a PDF of this page (on a Mac, simply select "print" and then select "print as PDF") before you submit it.

When you submit your (revised) paper to JMIR, please upload the PDF as supplementary file.

Don't worry if some text in the textboxes is cut off, as we still have the complete information in our database. Thank you!

Final step: Click submit !

Click submit so we have your answers in our database!

Pateikti

Niekada nepateikite slaptažodžių per „Google" formas.

[illegible] apie pažeidimą - Paslaugos sąlygos - Privatumo politika

Redaguojate savo atsakymą. Jei bendrinsite šį URL, ir kitiems žmonėms leisite redaguoti savo atsakymą.

UŽPILDYTI NAUJĄ ATSAKYMĄ

[illegible] formos
